# Sacubitril/Valsartan attenuates progression of diabetic cardiomyopathy through immunomodulation properties: an opportunity to prevent progressive disease

**DOI:** 10.1186/s12933-025-02741-5

**Published:** 2025-05-14

**Authors:** Narainrit Karuna, Lauren Kerrigan, Kevin Edgar, Mark Ledwidge, Ken McDonald, David J. Grieve, Chris J. Watson

**Affiliations:** 1 Wellcome-Wolfson Institute for Experimental Medicine, Queen’s University Belfast, Belfast, UK; 2 Department of Pharmaceutical Care, Faculty of Pharmacy, Chiang Mai University, Chiang Mai, Thailand; 3 STOP-HF Unit, St. Vincent’s University Healthcare Group and University College Dublin, Dublin, Ireland

**Keywords:** Diabetic cardiomyopathy, Sacubitril/Valsartan, IRF7, Immunomodulation, Inflammation

## Abstract

**Background and aims:**

Diabetic cardiomyopathy (DbCM) is recognised as a key mediator and determinant of heart failure (HF), particularly HF with preserved ejection fraction (HFpEF). Improved understanding of mechanisms underlying transition from early-stage DbCM to HFpEF will inform innovative evidence-based treatment approaches, which are urgently required to alleviate increasing disease burden. This study aimed to determine whether inhibition of neprilysin activity by Sacubitril/Valsartan in both experimental and clinical DbCM attenuates adverse remodelling through promotion of cardioprotective signalling.

**Methods and results:**

Sacubitril/Valsartan effectively reduced plasma neprilysin activity in both diabetic patients with pre-clinical HFpEF from the PARABLE trial (baseline (Val n = 25; Sac/Val n = 35) and 3 months after treatment (Val n = 21/25; Sac/Val n = 33/35)) and DbCM (high-fat diet and streptozotocin) mice. Plasma neprilysin activity at baseline was correlated with worsening cardiac performance at 18 months indicated by left atrial stiffness index in patients (n = 44/60), whilst diastolic dysfunction and pathological remodelling in DbCM mice were improved by Sacubitril/Valsartan, but not Valsartan. snRNA-sequencing showed that progressive experimental DbCM is characterised by chronic low-grade inflammation, reflected by increased infiltration of pro-inflammatory monocytes (Ccr2^+^ Ly6c^hi^) and reduction in MHC-II macrophages, which was prevented by Sacubitril/Valsartan. Informatics analysis implicated *IRF7* as a central mediator of Sacubitril/Valsartan-induced immunomodulation in DbCM, whilst treatment of M2-like pro-repair macrophages with the neprilysin inhibitor, LBQ657 and Valsartan suppressed glucose-induced *IRF7* expression and paracrine activation of cardiac fibroblast differentiation in vitro.

**Conclusion:**

Immune cells are significantly involved in DbCM progression, impacting myocardial homeostasis and HF progression. Neprilysin inhibition by Sacubitril/Valsartan improved adverse cardiac remodelling in experimental DbCM through direct regulation of inflammation, highlighting immunomodulation as a novel mechanism underlying established its cardioprotective actions.

**Graphical abstract:**

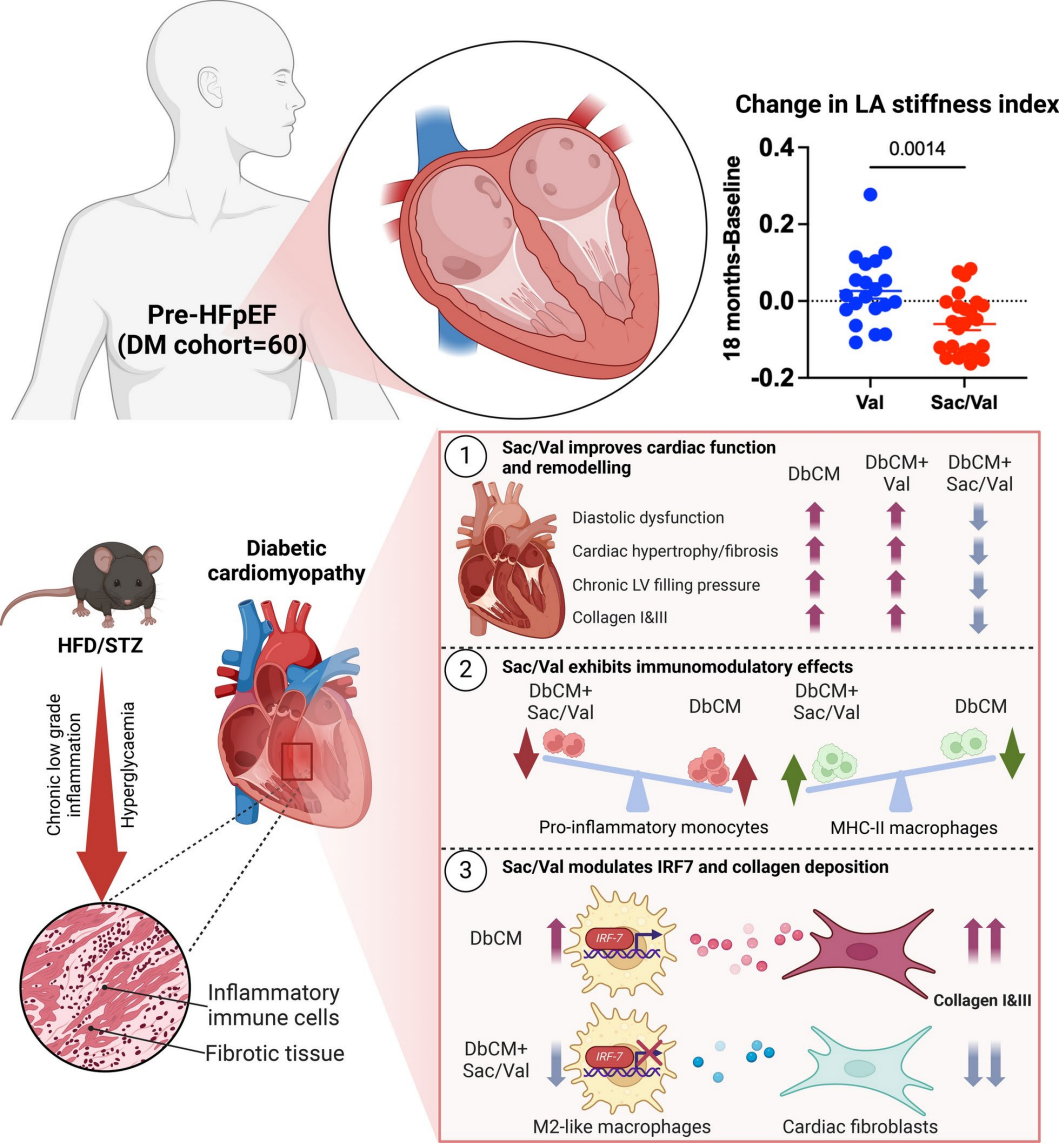

**Supplementary Information:**

The online version contains supplementary material available at 10.1186/s12933-025-02741-5.

## Introduction

Diabetes mellitus (DM), particularly type 2, is increasingly prevalent and a major global cause of morbidity and mortality [1, 2]. Epidemiological evidence linking DM and increased risk of heart failure (HF) is well established, with HF representing a significant proportion of cardiovascular disease (CVD) burden in DM patients [3, 4]. However, transition of at-risk individuals (Stage A HF) to symptomatic HF (Stage C HF) is not straightforward and typically involves multifaceted pre-clinical HF (Stage B HF) associated with progressive cardiac remodelling and functional impairment, which precedes onset of clinical symptoms [5]. Diabetic cardiomyopathy (DbCM) was originally considered as overt HF [6], but is now defined as Stage B HF, with high prevalence ranging from 11.7 to 67% in community-dwelling adults and recognised as a significant precursor for future symptomatic HF [7, 8]. Traditionally, DbCM is described as early diastolic dysfunction, potentially evolving into systolic dysfunction aggravated by hyperglycaemia and myocardial inflammation without other known cardiovascular disease (e.g., hypertension, coronary artery disease, atherosclerosis) [9–11]. Once diagnosed, DbCM is typically treated following standard HF approaches, which may be ineffective, with limited evidence-based therapeutic options. It is therefore critical to identify novel strategies which specifically target adverse remodelling underlying early-stage DbCM to more effectively prevent or delay progression to symptomatic HF whilst also reducing risk of other cardiac events.

Sacubitril/Valsartan is an angiotensin receptor neprilysin inhibitor (ARNI) that combines Valsartan, an established angiotensin receptor blocker with proven cardiac mortality and remodelling benefits in HF, with Sacubitril, a neprilysin (NEP) inhibitor providing additional protection [7, 12]. These additional impacts are primarily attributed to its complementary targeting of NEP, a zinc-activated endopeptidase that cleaves a large number of peptides, including natriuretic and insulinotropic peptides, which serve important roles in cardiovascular disease [13, 14]. In this regard, the recent PARABLE trial [15], which studied 250 patients with pre-HFpEF, reported that Sacubitril/Valsartan significantly reduced time to cardiovascular death and first major adverse cardiovascular event compared to Valsartan, providing the first direct evidence of benefit in asymptomatic pre-HF patients. Importantly, the findings of the PARABLE trial clearly highlight the value of further investigating specific use of Sacubitril/Valsartan in the pre-HF setting, including examination of phenotypic-specific subsets within this large heterogeneous at-risk population.

The aim of this study was, therefore, to interrogate detailed association of NEP activity with disease progression in pre-HFpEF patients with DM utilising both samples and data from the PARABLE trial [15], whilst undertaking complementary mechanistic studies in experimental DbCM to further understand therapeutic response to Sacubitril/Valsartan as a potential new evidence-based treatment option.

## Materials and methods

A comprehensive description of all materials and methods can be found in the Supplementary material online.

### Human pre-HFpEF cohort

A sub-cohort of patients with type 2 DM (n = 60/250) from the PARABLE trial (NCT04687111) was studied [15]. All eligible patients gave informed consent to be recruited to the study from April 2015 to June 2021, with inclusion and exclusion criteria as previously described [15]. Briefly, at time of study enrolment, patients were aged 40 years or older with pre-HFpEF identified by elevated natriuretic peptides and left atrial volume index (LAVI), and without history of symptomatic HF, left ventricular (LV) systolic dysfunction, serious valvular disease, or kidney dysfunction. Baseline characteristics of pre-HFpEF with type 2 DM are presented in Supplementary material online (Table S3). Correlation between baseline plasma NEP activity of type 2 DM pre-HFpEF patients and change in left atrial (LA) stiffness index at 18 months from baseline (n = 44/60) was assessed. Changes in plasma NEP activity between groups were investigated at baseline (Val n = 25; Sac/Val n = 35) and 3 months after treatment (Val n = 21/25; Sac/Val n = 33/35). Furthermore, we compared change in LA stiffness index at 18 months from baseline in Valsartan group (n = 20/25) and Sacubitril/Valsartan group (n = 24/35). The study was conducted according to the Declaration of Helsinki. The current study was reported in line with the STrengthening the Reporting of OBservational studies in Epidemiology (STROBE) guidelines (see Supplementary material online).

### Diabetic cardiomyopathy (DbCM) mouse model

An established DbCM mouse model was used combining high-fat diet (HFD) and single-dose streptozotocin (STZ; 100 mg/kg i.p.). Male C57BL/6J mice (8 weeks of age) were randomised to control diet (CD, n = 12) or HFD (n = 33) for 8 weeks prior to STZ or vehicle administration to promote metabolic and cardiac dysfunction. Mice remained on the study diets for 4 weeks before addition of Sacubitril/Valsartan (Sac/Val; 100 mg/kg/day) or Valsartan (Val; 50 mg/kg/day) to the drinking water of HFD/STZ groups with comparison to both HFD/STZ and CD groups, for a further 12 weeks. The 4 study groups were: CD mice (n = 12), HFD/STZ (n = 11), HFD/STZ + Sac/Val (n = 10), and HFD/STZ + Val (n = 12). Mice were housed under 12 h light/12 h dark cycle with access to diet and water ad libitum. Terminal analyses were performed at 24 weeks. Animal handling and all animal experiments were performed according to the guidelines from Directive 2010/63/EU of the European Parliament on the protection of animals used for scientific purposes and UK Home Office regulations and were approved by the local authorities.

### Echocardiography

Mice were anesthetised in a closed chamber with 3% isoflurane in oxygen and mice were in the supine position, and core temperature was maintained at 37 °C with 1.5% isoflurane in oxygen by nose cone during the procedure. LV dimensions and function were measured by 2-dimensional and Doppler echocardiography using a Vevo 3100 system (FUJIFILM VisualSonics). In addition, left atrial (LA) area and LA volume measurements were performed as previously described [16, 17].

### Morphometrics and histology

Mice were sacrificed at 24 weeks by cervical dislocation, and hearts were excised and weighed with normalisation to tibia length (TL). Heart tissue was fixed overnight in 10% neutral buffered formalin solution, with subsequent histological processing, paraffin embedding, and sectioning. All tissues were sectioned at 5 μm thickness using a microtome (Leica Biosystems) prior to staining with H&E (Haematoxylin and Eosin) and Picrosirius red for analysis of cardiomyocyte cross-sectional area and collagen deposition, respectively, followed by quantification using ImageJ (NIH, USA).

### Metabolic parameters

Mice were fasted for 4 h in the morning before tail vein blood samples were obtained for measurement of blood glucose (Glucomen® Areo; Menarini Diagnostics) and HbA1c (A1CNow kit; BHR Pharmaceuticals Ltd). Glucagon-like peptide-1 (GLP-1; 81508, Crystal Chem) and insulin (90080, Crystal Chem) levels were assessed by enzyme-linked immunosorbent assay (ELISA). Insulin sensitivity was evaluated by quantitative insulin sensitivity check index (QUICKI; 1/log fasting insulin [mU/L] + log fasting glucose [mg/dL]), whilst steady‐state β‐cell function (HOMA‐β) was calculated as 20 × fasting insulin (μIU/ml)/fasting glucose (mmol/ml) − 3.5.

### Quantitative real-time polymerase chain reaction (RT-qPCR) and Western blotting

Total RNA was extracted from cardiac tissue and cell pellets using TRIzol® reagent (Invitrogen™) and High Pure RNA Isolation Kit (11828665001, Roche), respectively. cDNA was produced using High-Capacity RNA-to-cDNA Kit (4387406; Thermo Fisher Scientific) and reverse transcription-quantitative polymerase chain reaction (RT-qPCR) performed using SYBR green master mix on a Roche LightCycler^®^ 480 platform with normalisation to Beta-2-Microglobulin. Primers were synthesised by Integrated DNA Technologies (Leuven, Belgium) with sequences included in the Supplementary material online (Table S1). Relative gene expression was calculated using the 2^−ΔΔCT^ method.

Protein was extracted, and concentrations were quantified using bicinchoninic acid assay (23227, Thermo Fisher Scientific) for Western blotting. Protein samples (20 µg) were separated by 12% SDS-PAGE prior to transfer to PVDF membranes (Immobilon™) and incubation with specific primary and secondary antibodies, detailed in the Supplementary material online (Table S2). Visualisation of protein bands was accomplished using Immobilon™ ECL Ultra Western HRP Substrate (WBULS0500, Merck) and expression quantified by ImageJ with normalisation to GAPDH.

### Single-nuclei RNA sequencing and downstream analysis

LV tissue from DbCM mice was subjected to single-nuclei RNA sequencing (snRNA-seq) to analyse cell-specific gene expression. Detailed information on snRNA-seq data processing, statistical comparison, and downstream analysis (pathway analysis, single-cell trajectory analysis, Gene Regulatory Network inference) is included in the Supplementary material online.

### In vitro studies

THP-1 cells, a human monocyte cell line, were obtained from the American Tissue Culture Collection (ATCC, Manassas, VA)were cultured in Roswell Park Memorial Institute medium without glucose (RPMI 1640, 10-043-CV, Corning) containing 10% heat-inactivated fetal bovine serum (FBS; Gibco), 10 mM Hepes (Merck), and 0.05 mM ß-mercaptoethanol (Merck) at 5% CO_2_ in 37 °C. THP-1 cells were differentiated into naïve macrophages (M0) using phorbol 12-myristate 13-acetate (PMA; Sigma, P8139) and incubated with IL4/IL13 (peproTech) in L-glucose (4.5 g/L) or d-glucose (4.5 g/L) RPMI medium to induce polarisation to M2-like pro-repair macrophages. Treatments (valsartan 1 μM, LBQ657 1 μM, valsartan + LBQ657 1 μM) were added for 24 h before cell harvesting and collection of conditioned media, which were passed through a 200 nm filter and stored at − 80 °C.

Specific small interfering RNA (siRNA) against *IRF7* (Horizon Discovery, UK) was transfected into M2-like macrophages (24 h) prior to collection of filtered conditioned media. Human cardiac fibroblasts (HCFs; ScienCell, Carlsbad, CA) were cultured in Dulbecco’s modified Eagle's minimal essential (DMEM) medium (Gibco) with low glucose (1 g/L) containing 2% FBS and treated with macrophage conditioned media (50% v/v) to assess paracrine influence on myofibroblast differentiation. In parallel, migration assay of HCFs treated with conditioned media from M2-like macrophage experiments was conducted at 0 h and 24 h. Cells were collected and stored at − 80 °C for subsequent analysis.

### Neprilysin activity

NEP activity was measured in homogenised LV tissue and plasma, as described by Pavo et al. [18]. with optimised adjustments. ZnCl_2_ was added to EDTA-plasma samples before mixing with Tris/HCl buffer for active wells or DL-Thiorphan (T6031, Merck Life Science) for reference wells, and reaction started by adding glutaryl-Ala-Ala-Phe-4-methoxy-2-naphthylamine (G3769, Merck). The reaction was stopped by adding DL-Thiorphan before addition of aminopeptidase M solution (164598, Merck) to generate methoxy-2-naphthylamine (fluorescence product). The fluorescence signal was read at 355/460 nm on a microplate reader (FLUOstar Omega, BMG LABTECH) with reference to a methoxy-2-naphthylamine (M9894, Merck) standard curve. NEP activity was calculated from the difference between active and reference wells to distinguish NEP activity from non-specific endopeptidase activity.

### Statistical analysis

R programme 4.3.1 with relevant packages and GraphPad Prism 10 (San Diego, CA, USA) were used for data analysis. All values are expressed as mean ± SD unless stated otherwise. Two groups were compared by Student’s t-test (paired or independent), Mann–Whitney U Test, or Wilcoxon matched-pairs signed rank test (paired samples), as appropriate. For comparison of more than 2 groups, one-way analysis of variance (ANOVA) followed by Tukey’s multiple comparison or Kruskal–Wallis followed by Dunn’s multiple comparison tests were used. A mixed ANOVA was used to compare mean differences between groups based on two factors. Analysis of covariance (ANCOVA) was used to compare changes in the dependent variable while adjusting for baseline variables with sequential models. Assumptions were tested prior to the analysis, and appropriate corrective measures were applied if any violations were found. Moreover, Spearman or Pearson correlation was applied as appropriate. All *P* values < 0.05 were considered as statistically significant.

## Results

### Neprilysin activity in clinical pre-heart failure

**Fig. 1:**
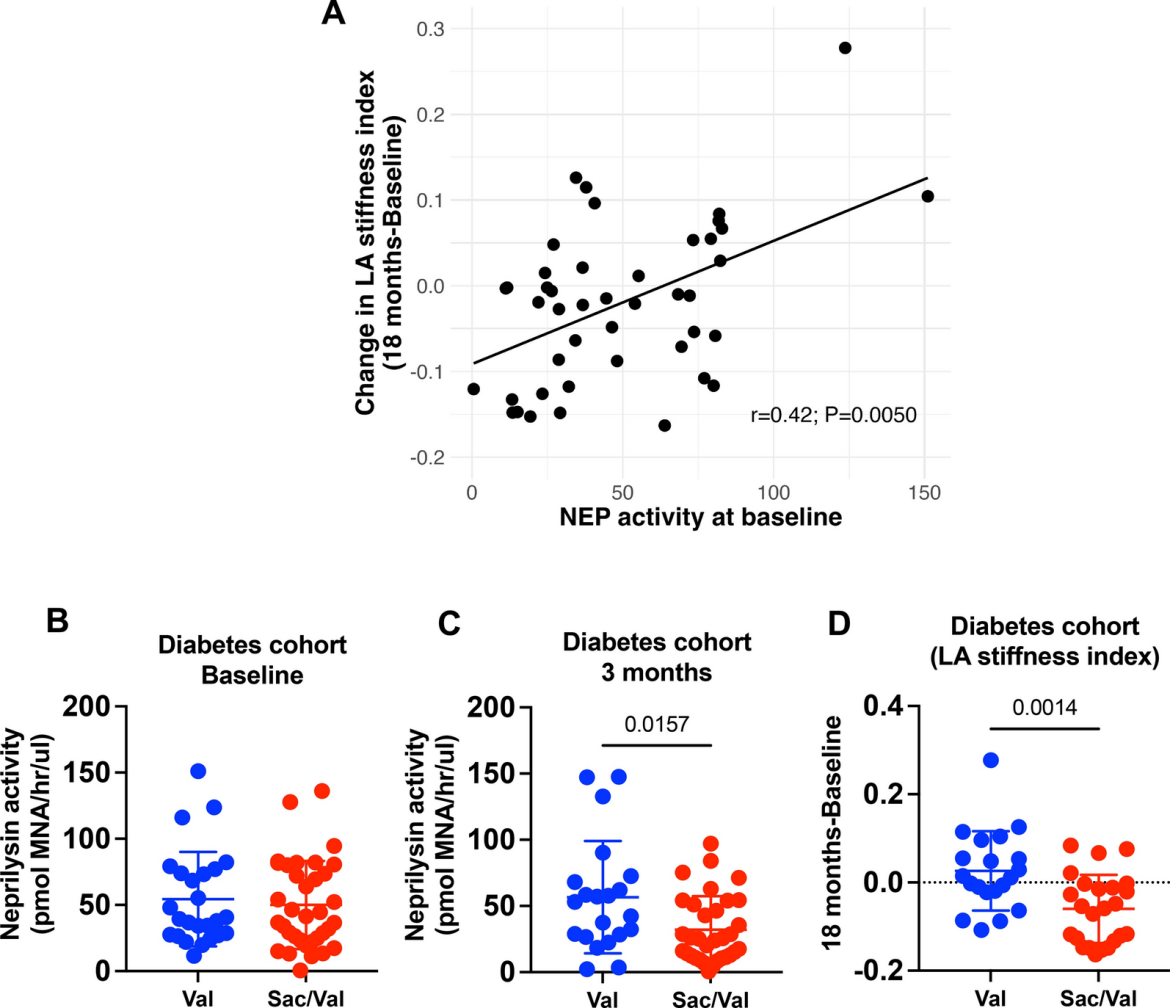
Plasma neprilysin activity and diastolic function markers in patients with diabetes. **A** Correlation analysis between plasma NEP activity of patients with diabetes at baseline and change in LA stiffness index (18 months-Baseline; n = 44/60). **B**, **C** Plasma neprilysin activity of pre-HFpEF patients with diabetes from the PARABLE trial at baseline (Val n = 25; Sac/Val n = 35) and 3 months after treatment (Val n = 21/25; Sac/Val n = 33/35). **D** Change LA stiffness index at 18 months from baseline in Valsartan group (n = 20/25) and Sacubitril/Valsartan group (n = 24/35). n = Patients (biological replicates). Correlation using Spearman’s rank-order correlation test (**A**) and comparison using either Mann–Whitney test (**B**, **C**) or Unpaired *t* test (**D**). If *P* values > 0.05 are not shown in graphs. LA = Left atrial; Sac/Val = Sacubitril/valsartan; Val = Valsartan

Activity of NEP enzyme, the primary target of Sacubitril, was quantified in plasma of a sub-cohort of pre-HFpEF patients with DM from the PARABLE trial [15] (n = 60/250) to understand impact of Sacubitril/Valsartan in this specific population. Type 2 DM patients (n = 44/60) showed positive correlation between plasma NEP activity at baseline and change in LA stiffness index at 18 months (r = 0.42, *P* = 0.0050; Fig. 1A), indicating clear relationship with cardiac remodelling. The significant relationship between LA stiffness index changes and baseline plasma NEP activity (Fig. 1A) remained significant even after adjusting age, gender, baseline B-type natriuretic peptide (BNP), and baseline LA stiffness index (Model 1; *P* = 0.0080). This association persisted after further adjustment for Model 1 with hypertension, obesity, coronary artery disease (CAD), chronic kidney disease (CKD), atrial fibrillation (AF), and other arrhythmias (Model 2; *P* = 0.0053). Prior to treatment, plasma NEP activity was not different between Sacubitril/Valsartan and Valsartan groups but was decreased in the Sacubitril/Valsartan group after 3 months (*P* = 0.0157*;* Val n = 25; Sac/Val n = 35 at baseline and Val n = 21/25; Sac/Val n = 33/35 at 3 months after treatment; Fig. 1B, C). Notably, the higher plasma NEP activity at 3 months in Valsartan versus Sacubitril/Valsartan remained significant with adjustment for age, gender, baseline BNP, and baseline NEP activity (Model 1; *P* = 0.0046; Fig. 1C). This difference remained significant in Model 2, which further adjusted for hypertension, obesity, CAD, CKD, AF, and other arrhythmias (*P* = 0.0002, Fig. 1C). Furthermore, diabetic patients receiving Sacubitril/Valsartan showed decreased LA stiffness index at 18 months from baseline, compared to those in the Valsartan group (*P* = 0.0014; Val n = 20/25; Sac/Val n = 24/35; Fig. 1D) even with adjustment for age, gender, baseline BNP, and baseline LA stiffness index (Model 1; *P* = 0.0100). This difference persisted after further adjustment for Model 1 with hypertension, obesity, CAD, CKD, AF, and other arrhythmias (Model 2; *P* = 0.0360). These important initial clinical data suggest that early suppression of NEP activity could be significant in preventing or delaying HF progression in patients with DM. We, therefore, hypothesised that NEP inhibition may attenuate adverse cardiac structural and functional remodelling associated with DbCM. Therefore, we proceeded to conduct detailed mechanistic analysis using an established mouse model that displays typical clinical features of DbCM, characterised by diastolic dysfunction and cardiac hypertrophy with preserved systolic function.

### DbCM mice treated with Sacubitril/Valsartan, but not Valsartan, demonstrate improved diastolic function and preserved cardiac structure

**Fig. 2:**
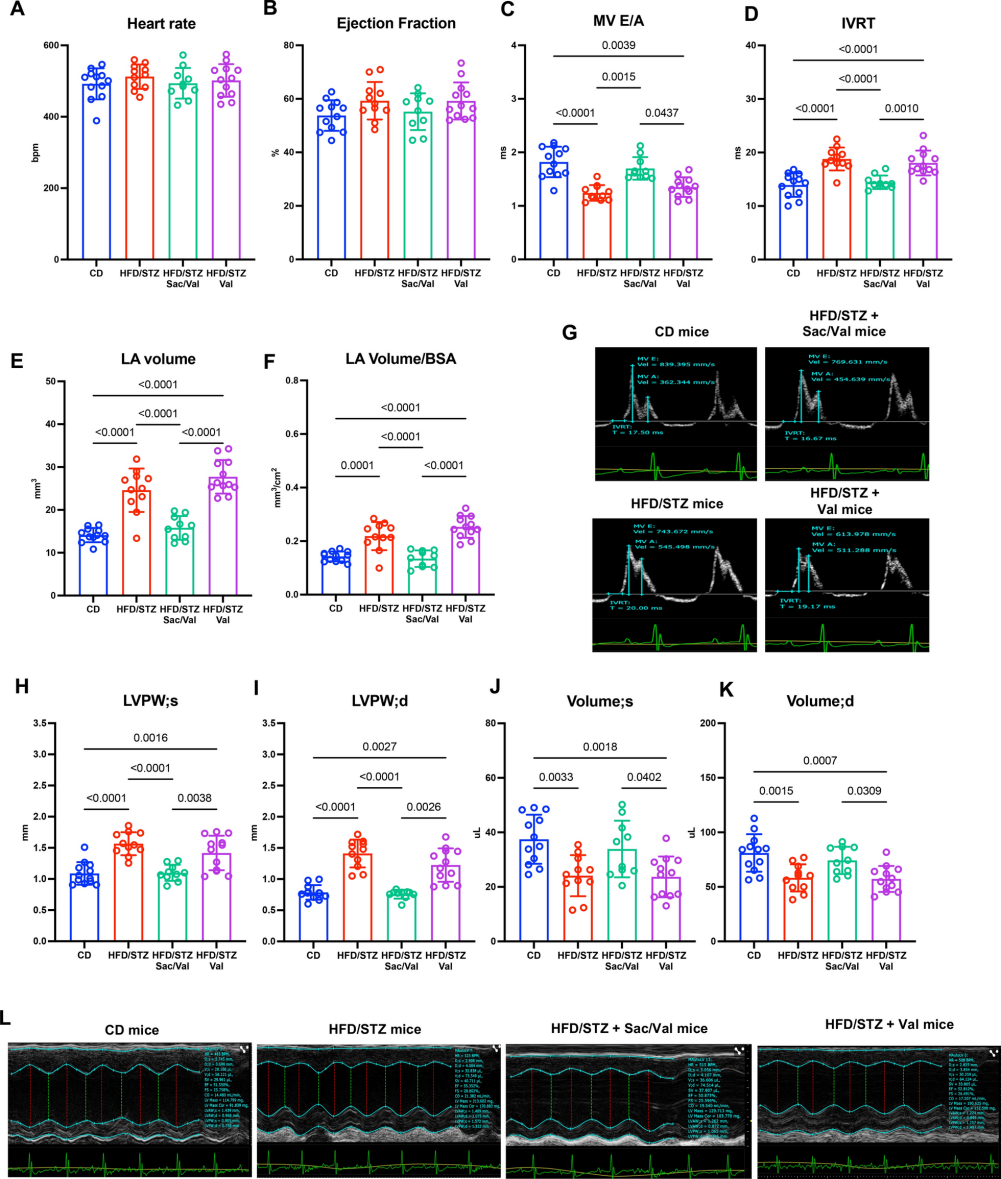
Measurement of left ventricular function and structure at 24 weeks of study by echocardiography. **A**–**F** Mice are evaluated for cardiac function and disease progression using echocardiography. **G** Representative images of ratio of MV E/A using PW-doppler. **H**–**K** Mice are evaluated for cardiac structure. (L) Representative images of left ventricular structure using M-mode. CD n = 12; HFD/STZ n = 11; HFD/STZ + Sac/Val n = 10; HFD/STZ + Val n = 12. n = Number of mice (biological replicates). Comparison using either one-way ANOVA followed by Tukey's multiple comparisons test (**A**, **B**, **D**, **E**–**F**, **H**, **J**, **K**) or Kruskal–Wallis test followed by Dunn's multiple comparisons test (**C**, **I**). *P* values for one-way ANOVA/Kruskal–Wallis test < 0.05 for (**C**–**F**) and (**H**–**K**). If *P* values > 0.05 are not shown in graphs. BPM = Beat per minute; CD = Control diet; HFD = High-fat diet; STZ = Streptozotocin; BPM = Beat per minute; MV E/A = Ratio of E-wave velocity to A-wave velocity; IVRT = Isovolumic relaxation time; LA = Left atrial; BSA = Body surface area; LVPW = Left ventricular posterior wall. Other abbreviations as in Fig. 1

**Fig. 3:**
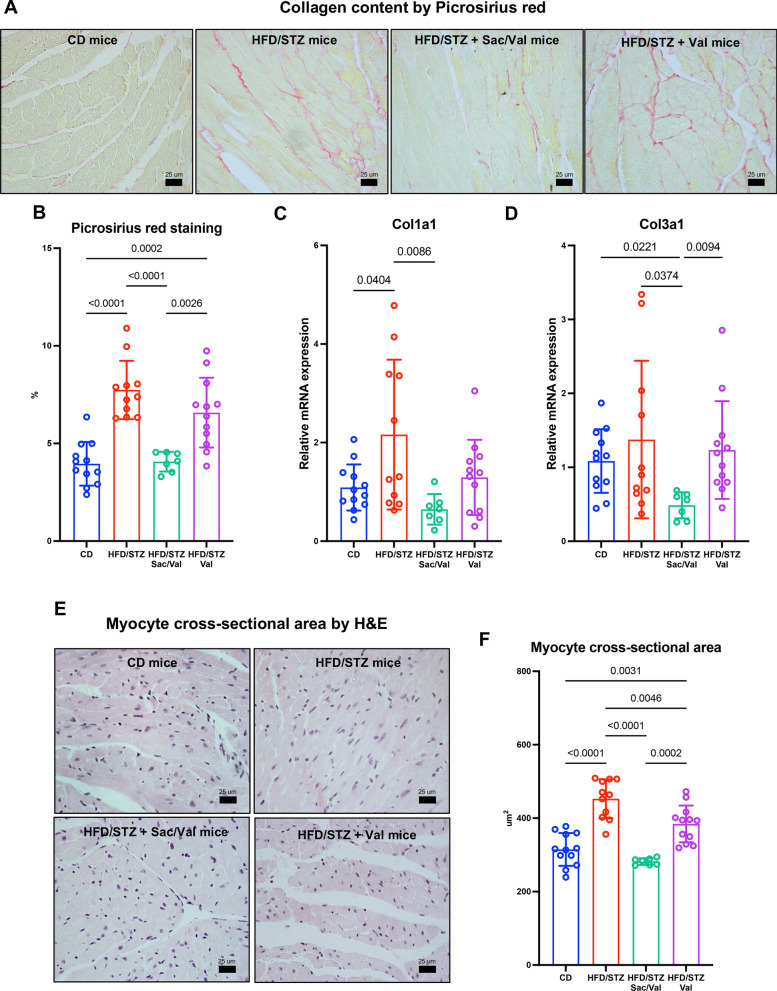
Histology studies and assessment of pro-fibrotic genes and cardiac remodelling. **A** Representative photomicrographs of myocardium stained with Picrosirius red for interstitial fibrosis. **B** Quantification of collagen content from picrosirius red staining (CD n = 12; HFD/STZ n = 11; HFD/STZ + Sac/Val n = 7; HFD/STZ + Val n = 12). **C**, **D** Relative mRNA expression of collagen 1a1 and 3a1 (CD n = 12; HFD/STZ n = 11; HFD/STZ + Sac/Val n = 7; HFD/STZ + Val n = 12), respectively. **E** Representative photomicrographs of cardiomyocyte cross sectional area stained with Haematoxylin and Eosin. **F** Quantification of cardiomyocyte cross sectional area from Haematoxylin and Eosin (CD n = 12; HFD/STZ n = 11; HFD/STZ + Sac/Val n = 7; HFD/STZ + Val n = 12). n = Number of mice (biological replicates). Comparison using either one-way ANOVA followed by Tukey's multiple comparisons test (**B**–**C**, **F**) or Kruskal–Wallis test followed by Dunn's multiple comparisons test (**D**). *P* values for one-way ANOVA/Kruskal–Wallis test < 0.05 for (**B**–**D**), and (**F**). If *P* values > 0.05 are not shown in graphs. Abbreviations as in Figs. 1 and 2

HFD/STZ mice were allocated to Sacubitril/Valsartan (100 mg/kg/day) and Valsartan (50 mg/kg/day) groups with treatments titrated based on daily water consumption to achieve target doses which were maintained throughout the 12-week treatment period (Supplementary Figure S1A-B) prior to analysis of cardiac structure and function by echocardiography. Heart rate and ejection fraction were not different between groups (Fig. 2A, B). However, MV E/A ratio was considerably decreased in both HFD/STZ and Valsartan groups, compared to control group, indicating impaired diastolic function, which was restored by Sacubitril/Valsartan treatment (Fig. 2C, G). Importantly, longitudinal follow-up echocardiography revealed that MV E/A ratio was the first diastolic marker to recover after 4 weeks of Sacubitril/Valsartan, and IVRT decreased after 8 weeks of Sacubitril/Valsartan, with maintained benefit over the 12-week treatment period (Supplementary Figure S3). These cardioprotective effects of Sacubitril/Valsartan were also reflected in IVRT, which was prolonged in HFD/STZ and Valsartan groups compared to control mice and restored with Sacubitril/Valsartan treatment (Fig. 2D, G). Similarly, both absolute and normalised LA volume, as indicators of chronic LV filling pressure, were increased in HFD/STZ and Valsartan groups, compared to controls, whilst HFD/STZ mice treated with Sacubitril/Valsartan showed reduced LA volume (Fig. 2E, F). In addition, echocardiography revealed that HFD/STZ mice developed LV hypertrophy and remodelling compared to control mice, reflected by increased wall thickness and systolic/diastolic volumes, which were normalised by treatment with Sacubitril/Valsartan but not with Valsartan (Fig. 2H, L). Picrosirius red staining of cardiac tissue indicated that HFD/STZ mice treated with Sacubitril/Valsartan had lower collagen content than HFD/STZ and Valsartan groups (Fig. 3A,B) together with reduced mRNA expression of pro-fibrotic genes (*Col1a1* and *Col3a1*; Fig. 3C, D), whilst cardiomyocyte cross-sectional area was decreased in Sacubitril/Valsartan versus Valsartan and HFD/STZ groups (Fig. 3E, F). Taken together, these data show clear benefits of Sacubitril/Valsartan against diastolic dysfunction and adverse cardiac remodelling associated with experimental DbCM, which are not evident with Valsartan alone as pivotal pathogenic factors underlying disease progression.

### Increased plasma NEP activity in experimental DbCM is inhibited by Sacubitril/Valsartan and associated with improvement of echocardiographic parameters

**Fig. 4:**
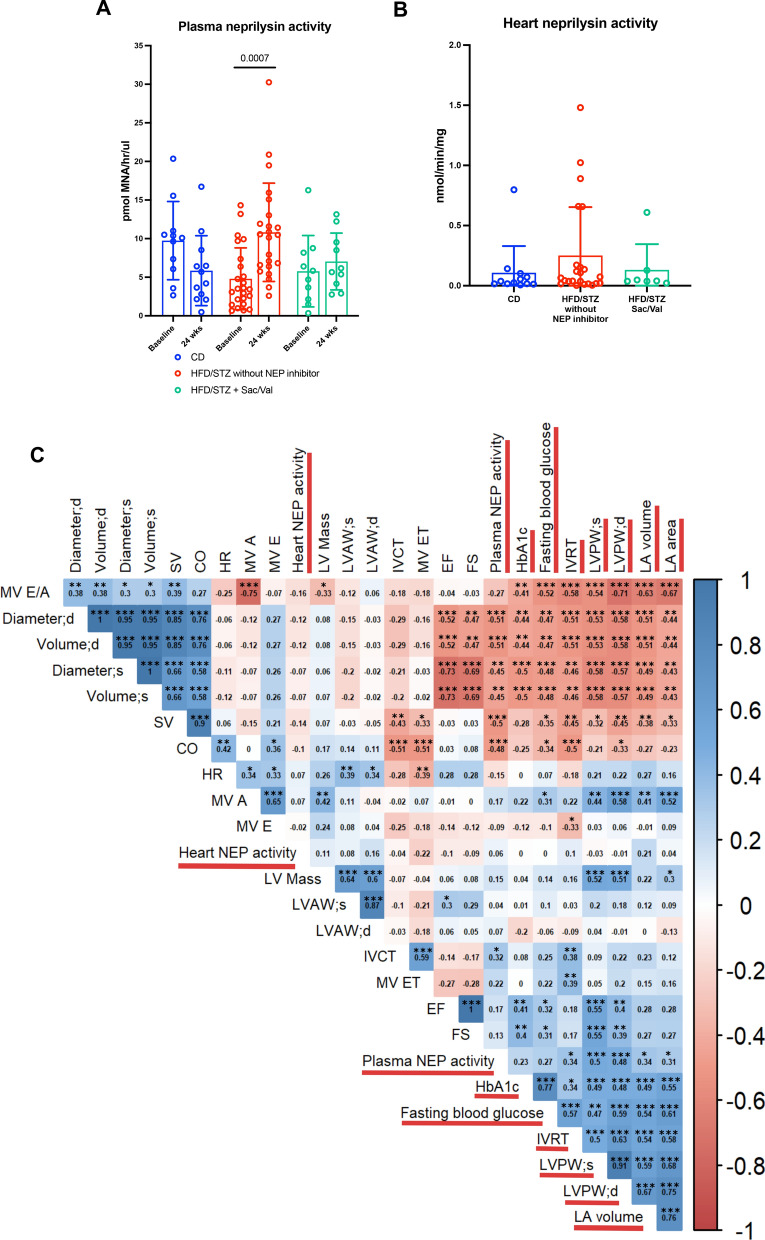
Measurement of neprilysin activity and correlations among echocardiographic parameters. **A** Plasma neprilysin activity of control mice (n = 11–12), HFD/STZ mice without NEP inhibitor (n = 23–24), and HFD/STZ + Sac/Val mice (n = 10) at baseline and 24 weeks of study. **B** Heart neprilysin activity across different groups (CD n = 12; HFD/STZ without NEP inhibitor n = 23; HFD/STZ + Sac/Val n = 7) at 24 weeks of study. **C** Correlation matrix among echocardiography parameters, neprilysin activity, and diabetic parameters at 24 weeks of study (CD n = 12; HFD/STZ n = 11; HFD + Sac/Val n = 7–10; HFD/STZ + Val n = 12). n = Number of mice (biological replicates). Comparison using Paired *t* test or Wilcoxon matched-pairs signed rank test (**A**). Comparison using Kruskal–Wallis test followed by Dunn's multiple comparisons test (**B**). Correlation using Spearman’s rank-order correlation test (**C**). If *P* values > 0.05 are not shown in graphs. NEP = Neprilysin; SV = Stroke volume; CO = Cardiac output; HR = Heart rate; LVAW = Left ventricular anterior wall; IVCT = Isovolumic contraction time; EF = Ejection fraction; FS = Fraction shortening; HbA1c = Haemoglobin a1c. Other abbreviations as in Fig. 1–2. *P < 0.05; **P ≤ 0.01; ***P ≤ 0.001

Plasma NEP activity was measured at baseline and 24-week study endpoint. Sacubitril/Valsartan effectively inhibited increases in plasma NEP activity observed in HFD/STZ without NEP inhibitor mice, compared to baseline (Fig. 4A), whilst heart NEP activity levels were not different across groups at the end of the study (Fig. 4B). The relationship between NEP activity and echocardiography parameters was examined using the Spearman correlation coefficient, which revealed that plasma NEP activity, rather than heart NEP activity, exhibited positive correlations with several cardiac parameters: LVPW;s (r = 0.50, *P* = 0.0004), LVPW;d (r = 0.48, *P* = 0.0008), LA volume (r = 0.34, *P* = 0.0227), LA area (r = 0.31, *P* = 0.0384), and IVRT (r = 0.34, *P* = 0.0206) (Fig. 4C). These findings suggest that elevated circulating NEP activity is linked to worsened cardiac structural and functional remodelling, including diastolic dysfunction (prolonged IVRT), elevated LV filling pressure (increased LA area and volume) and cardiac hypertrophy (enlarged LVPW;s and LVPW;d). Altogether, these data suggest that inhibition of circulating NEP activity by Sacubitril/Valsartan at an early stage of disease may impede progression of DbCM.

### DbCM mice treated with Sacubitril/Valsartan show improvement in glycaemic parameters but not insulin sensitivity

**Fig. 5:**
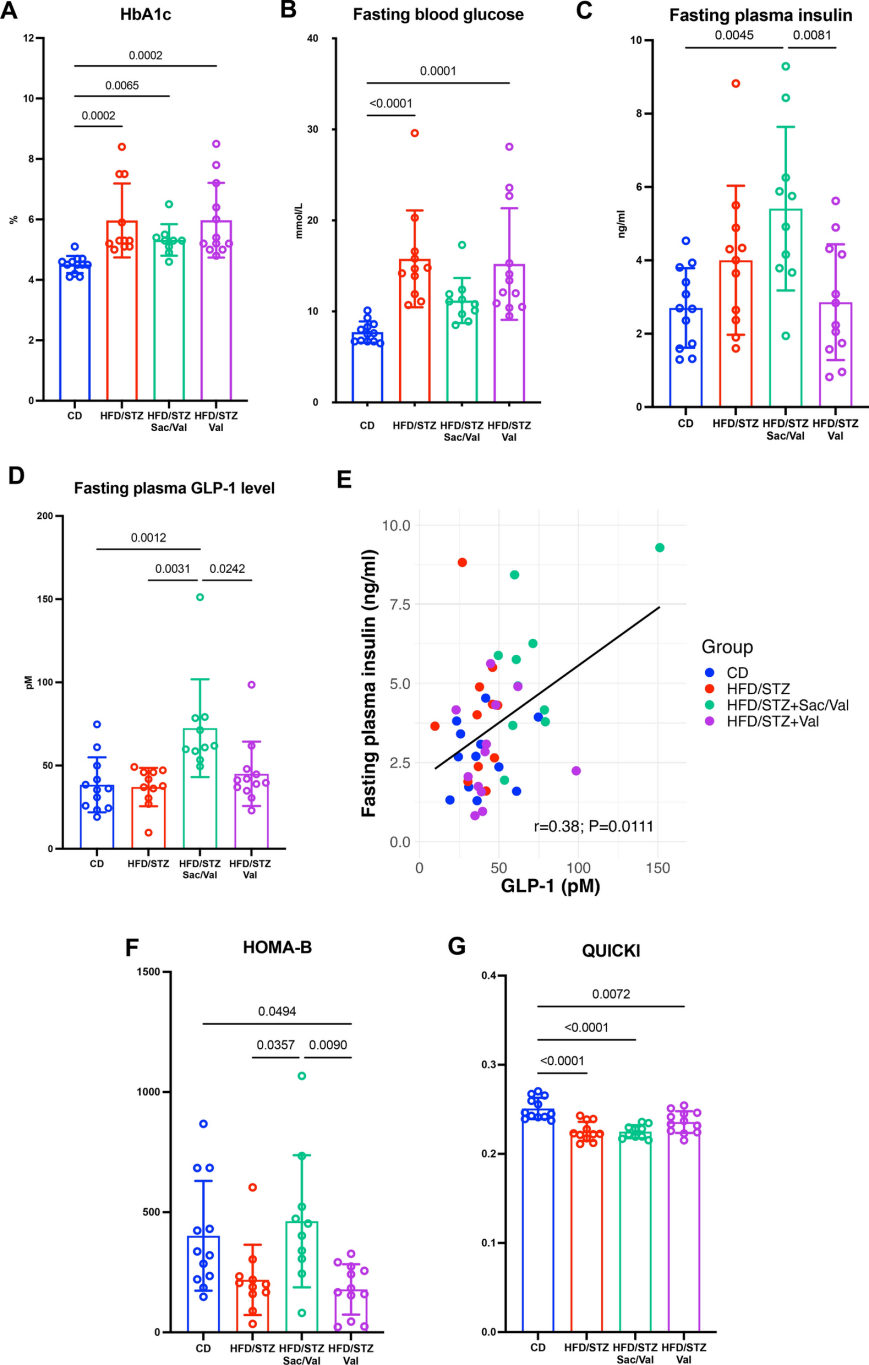
Measurement of diabetic parameters. **A**, **B** HbA1c and fasting blood glucose levels at 24 weeks of study. **C**, **D** Fasting plasma insulin and GLP-1 levels at 24 weeks of study. **E** Correlation between GLP-1 and fasting plasma insulin (n = 45). **F**, **G** Assessment of HOMA-B and QUICKI levels across groups. CD n = 12; HFD/STZ n = 11; HFD/STZ + Sac/Val n = 10; HFD/STZ + Val n = 12. n = Number of mice (biological replicates). Comparison using either one-way ANOVA followed by Tukey's multiple comparisons test (**C**, **F**–**G**) or Kruskal–Wallis test followed by Dunn's multiple comparisons test (**A**, **B**, **D**). Correlation using Spearman’s rank-order correlation test (**E**). *P* values for one-way ANOVA/Kruskal–Wallis test < 0.05 for (**A**–**D**), and (**F**–**G**). If *P* values > 0.05 are not shown in graphs. GLP-1 = Glucagon-Like peptide-1; HOMA-B = Homeostatic model assessment B-cell function; QUICKI = Quantitative insulin sensitivity check index. Other abbreviations as in Figs. 1 and 2

HFD/STZ mice developed a DM-like metabolic phenotype characterised by elevated HbA1c and fasting blood glucose (FBG; Fig. 5A, B), associated with cardiac structural and functional impairment (Fig. 4C). Interestingly, FBG was improved with Sacubitril/Valsartan treatment in parallel with associated changes in LV diastolic function (MV E/A and IVRT), HF progression (LA volume and LA area), and LV hypertrophy (LVPW;s and LVPW;d) (Figs. 2, 5B). Fasting plasma insulin and GLP-1 (established NEP substrates [14]) were examined to underpin improved FBG by NEP inhibition. Indeed, fasting plasma insulin was higher in the Sacubitril/Valsartan group compared to the Valsartan (*P* = *0.0081*) and control (*P* = *0.0045*) groups (Fig. 5C), whilst GLP-1 was higher in the Sacubitril/Valsartan group, compared to control (*P* = 0.0012), HFD/STZ (*P* = 0.0031) and Valsartan groups (*P* = 0.0242) (Fig. 5D). Moreover, fasting plasma insulin was positively correlated with GLP-1 levels (r = 0.38, *P* = 0.0111; Fig. 5E). HOMA‐β score was highest in the Sacubitril/Valsartan group and comparable to control mice, although equivalent improvement in QUICKI score as a marker of insulin sensitivity was not observed (Fig. 5F, G and Supplementary Table S4). These results indicate that Sacubitril/Valsartan augments plasma GLP-1 and insulin levels and improves FBG and beta-cell function in experimental DbCM but does not affect insulin sensitivity, in parallel with beneficial impacts on cardiac structure and function.

### Single-nuclei RNA sequencing reveals differentially impacted cell populations in DbCM mouse hearts

**Fig. 6:**
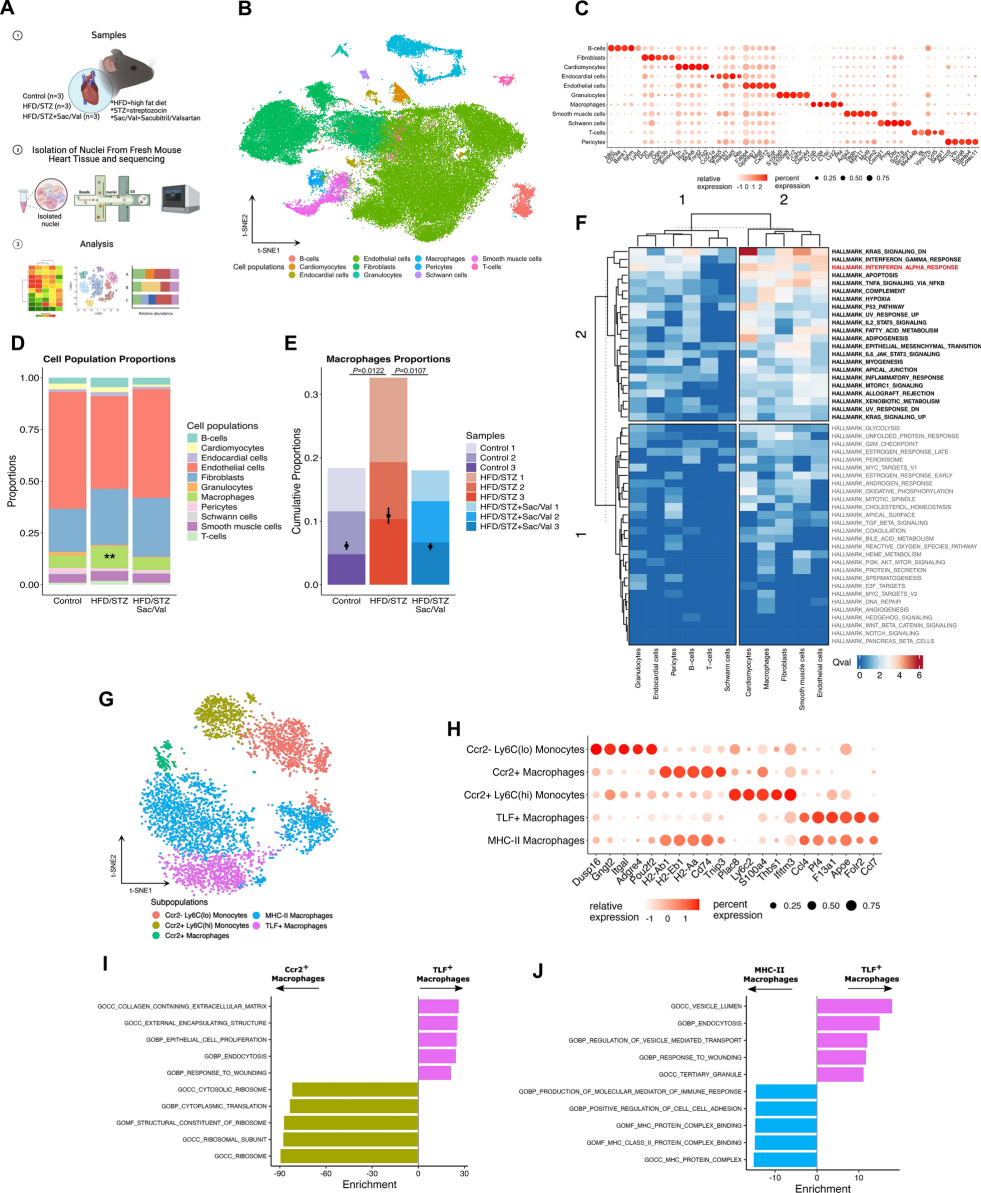
Single-nuclei RNA Sequencing (snRNA-seq) of DbCM mouse model. **A** Experimental design of snRNA-seq (control, HFD/STZ, and HFD/STZ + Sac/Val; n = 3 each). Images rendered by Biorender.com. Image usage is covered by BioRender’s Academic License Terms. **B** t-SNE dimensionality reduction of 11 major cell populations of mouse cardiac tissue. **C** Top 5 upregulated genes for each cell population where colour indicates strength of expression and size of dot represents percentage of cells expressing the gene. **D** Relative abundance of each cell population for sample groups (control, HFD/STZ, and HFD/STZ + Sac/Val; n = 3 each). **E** Relative macrophages abundance in each sample and group (control, HFD/STZ, and HFD/STZ + Sac/Val; n = 3 each). **F** Pathway analysis comparing between HFD/STZ mice and control mice. **G** Subpopulations of macrophages/monocytes. (H) Top 5 upregulated genes for Subpopulations of macrophages/monocytes where colour indicates strength of expression and size of dot represents percentage of cells expressing the gene. **I**, **J** Differentially expressed genes in TLF^+^ macrophages were compared to the Ccr2^+^ macrophages and the MHC-II macrophages. n = Number of mice (biological replicates). Comparison using one-way ANOVA (**D**) and Unpaired *t* test (**E**). *P* values for one-way ANOVA test < 0.05 for (**D**). If *P* values > 0.05 are not shown in graphs. Qval indicates the size of distribution change for pathways. Enrichment = Fold changes. Other abbreviations as in Figs. 1 and 2. **P ≤ 0.01

snRNA-seq was undertaken to define the roles of specific cardiac cell populations in DbCM progression and elucidate modes of action of Sacubitril/Valsartan treatment, using heart tissue from control, HFD/STZ, and HFD/STZ + Sac/Val mice (n = 3 each; Fig. 6A). After pre-processing and quality control filtering, data manifolds were constructed using t-distributed stochastic neighbour embedding (t-SNE), with comparison of 21,333 high-quality nuclei from control mice, 15,517 from HFD/STZ mice and 25,831 from HFD/STZ mice treated with Sacubitril/Valsartan. Cell populations were annotated after dimensionality reduction and graph-based clustering, which identified 11 distinct groups expressing known markers of major cardiac cell types based on previous publications (Fig. 6B) [19–22]. These clusters comprised B-cells (*Cd79a*, *Cd79b*, *Ms4a1*), cardiomyocytes (*Myh6*, *Tnnt2*, *Ttn*), endocardial cells (*Npr3*), endothelial cells (*Cdh5*, *Pecam1*, *Emcn*), fibroblasts (*Pdgfra*, *Col1a1*), granulocytes (*Ccr1*, *Csf3r*, *S100a9*), macrophages (*Fcgr1*, *Adgre1*), pericytes (*Kcnj8*, *Vtn*), schwann cells (*Plp1*, *Cnp*), smooth muscle cells (*Tagln*, *Myh11*), and T-cells (*Cd3e*, *Cd3d*, *Lef1*) (Supplementary Figure S4). Top enriched genes and specifically marked genes in each cell population revealed a diverse range of cell types across all samples (Fig. 6C).

Compositional analysis identified different proportions of cell populations in each group (Fig. 6D). The proportions of individual samples are presented in Supplementary Figure S5. Using arcsin square root transformation of proportions to compare abundance of each cell population, only the macrophage population significantly changed in abundance using one-way ANOVA (*P* = 0.0091), as shown in Fig. 6D. Comparison of macrophage abundance amongst sample groups showed that hearts of HFD/STZ mice had higher cell numbers than control mice (*P* = 0.0122), which was suppressed in the Sacubitril/Valsartan group compared to HFD/STZ mice (*P* = 0.0107; (Fig. 6E). These data suggest that macrophages may be one of principal contributors to DbCM progression, which are preferentially impacted by Sacubitril/Valsartan.

Further systems-level characterisation of pathway perturbations performed in HFD/STZ mice versus control mice indicated significant dysregulation of key DbCM pathways in macrophages and other cell populations (Fig. 6F). These pathways included well-defined immunological signatures responding to inflammation triggers, including type 1 interferon response pathway (interferon alpha response; Fig. 6F). Collectively, these data suggest that macrophages are one of the key drivers involved in progression of DbCM through modulation of inflammatory responses and that Sacubitril/Valsartan may specifically target macrophage migration and signalling to protect against diabetes-induced cardiac dysfunction.

### Roles of cardiac macrophage/monocyte subsets

The heart contains heterogeneous populations of macrophages/monocytes [23–25] which orchestrate homeostatic, inflammatory, and reparative processes [26]. Sub-clustering using unbiased snRNA-seq was employed to identify macrophage/monocyte subpopulations (Fig. 6G). Based on expression of C–C motif chemokine receptor 2 (*Ccr2*) and T cell immunoglobulin and mucin domain containing 4 (*Timd4*), these cells were categorised as Ccr2^+^ recruited macrophages or Timd4^+^ Ccr2^-^ resident macrophages (Supplementary Figure S6A). In detail, resident macrophages were denoted as TLF^+^ macrophages (cluster 5) with combination of specifically expressed *Timd4*, lymphatic vessel endothelial hyaluronan receptor 1 (*Lyve1*), folate receptor beta (*Folr2*), scavenger receptor cysteine-rich type 1 protein M130 (*Cd163*), and C–C motif chemokine ligand 24 (*Ccl24*), whereas Ccr2 ^+^ macrophages (cluster 7) were identified based on high expression of the *Ccr2* gene and high levels of major histocompatibility complex II (MHC-II) genes (*H2-Ab1* and *H2-Eb1*). The majority of macrophage subpopulations lacked expression of *Timd4*, *Lyve1*, *Folr2*, and *Ccr2,* but highly expressed MHC-II genes were denoted as MHC-II macrophages (cluster 1, 4, 8, and 9). Monocyte subpopulations were classified based on expression of lymphocyte antigen 6 complex, locus C2 (*Ly6c2*) and placenta specific 8 (*Plac8*) in addition to *Ccr2* gene. Our dataset showed 2 distinct monocyte subpopulations, including Ccr2^-^ Ly6c^lo^ monocytes (cluster 2 and 6) and Ccr2^+^ Ly6c^hi^ monocytes (cluster 3). The top 5 distinct genes for each subpopulation were identified in an unbiased fashion, as illustrated in Fig. 6H.

Cluster-defining gene expression was plotted as a function of pseudotime to track changes across different macrophage/monocyte states (Supplementary Figure S6B). TLF^+^ macrophages expressed *Timd4*, *Lyve1*, *Folr2, Cd163*, *Ccl24* and lacked expression of *Ccr2*, and MHC-II genes. Both MHC-II and Ccr2^+^ macrophages highly expressed *H2-Ab1* and *H2-Eb1* (MHC-II genes) but showed lower expression of signature genes of TLF^+^ macrophages and only Ccr2^+^ macrophages highly expressed in *Ccr2* gene. Expression of *Ccr2* and *Ly6c2* identified 2 distinct monocyte subpopulations (Ccr2^-^ Ly6c^lo^ and Ccr2^+^ Ly6c^hi^), with *Plac8* showing higher expression in Ccr2^+^ Ly6c^hi^ monocytes. These data suggest that tracking specific gene expression across macrophages and monocytes reveals unique subpopulations in heart tissue.

Pathway analysis of TLF^+^ macrophages compared with other macrophage subpopulations highlighted involvement in homeostatic and reparative activities, including collagen-containing extracellular matrix (ECM), response to wounding, endocytosis, and proliferation (Fig. 6I, J). Ccr2^+^ macrophages and MHC-II macrophages, when compared with TLF^+^ macrophages, were strongly implicated in ribosomal pathways and antigen-processing MHC pathways, respectively (Fig. 6I-J), whilst Ccr2^+^ Ly6c^hi^ monocytes were associated with classic inflammatory pathways, including immune effector process, response to bacterium, and cytokine production, relative to Ccr2^-^ Ly6c^lo^ monocytes (Supplementary Figure S7). Together, these analyses indicate that specific subpopulations of macrophages and monocytes serve distinct and distinctive functions and roles in DbCM hearts.

### Sacubitril/Valsartan affects abundance of cardiac macrophage/monocyte subsets in DbCM

**Fig. 7:**
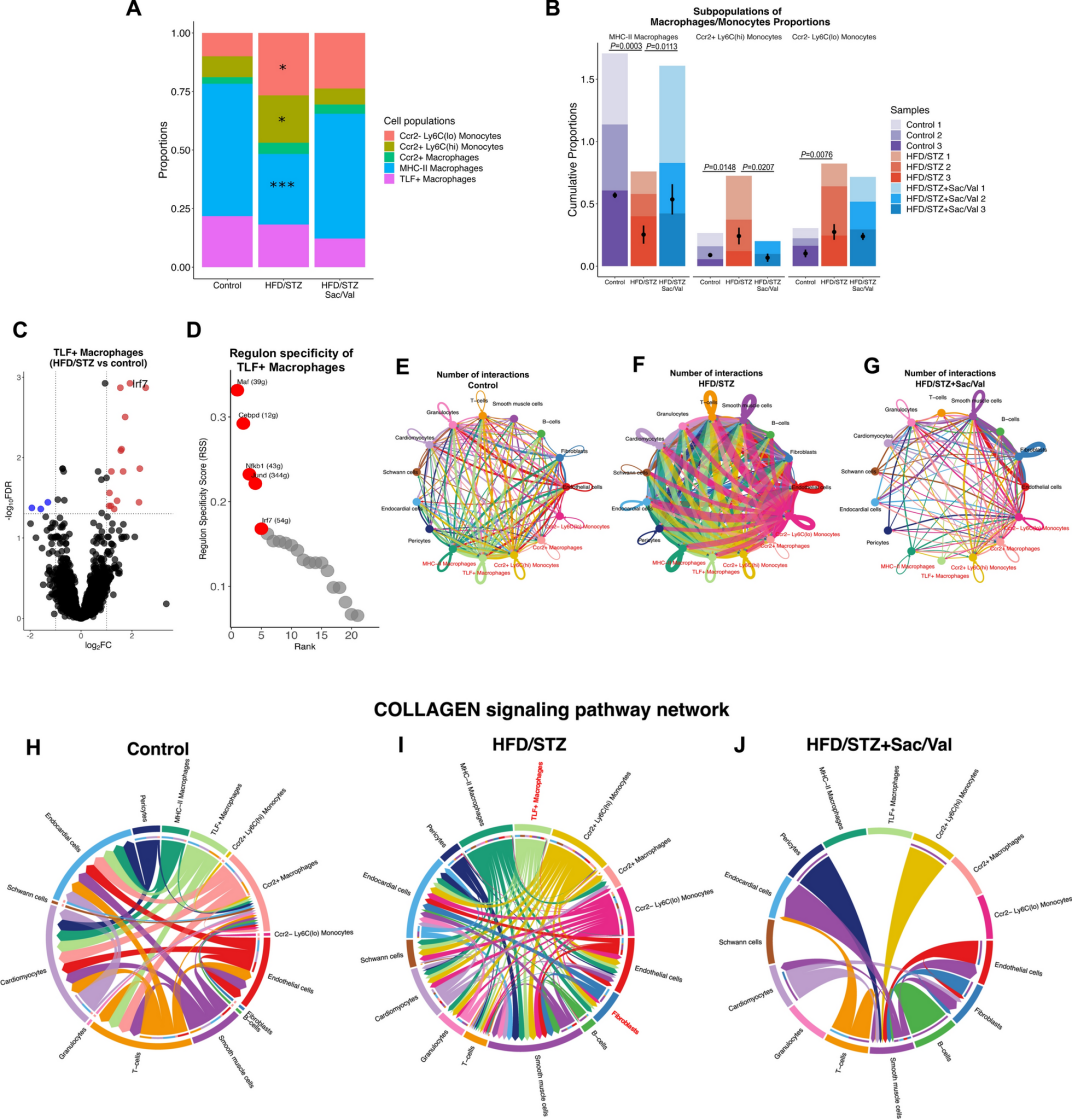
Abundance of subpopulations of macrophages/monocytes, roles of *Irf7* gene in DbCM and cell–cell interaction studies. **A** Relative abundance of each subpopulation of cell macrophages/monocytes for sample groups (control, HFD/STZ, and HFD/STZ + Sac/Val; n = 3 each). **B** Relative subpopulation of cell macrophages/monocytes abundance in each sample and group (control, HFD/STZ, and HFD/STZ + Sac/Val; n = 3 each). **C** Differential gene expression in TLF^+^ macrophages (HFD/STZ mice vs Control mice; n = 3 each). **D** Regulon specificity of TLF^+^ macrophages. **E**–**G** Possible interactions between all cell populations across sample groups. **H**–**J** Chord diagrams of inferred COLLAGEN signalling pathway networks across sample groups (control, HFD/STZ, and HFD/STZ + Sac/Val; n = 3 each). n = Number of mice (biological replicates). Comparison using one-way ANOVA (**A**) and Unpaired *t* test (**B**). *P* values for one-way ANOVA test < 0.05 for (**A**, **B**). If *P* values > 0.05 are not shown in graphs. Edge colour is consistent with the signalling source. Segments with large arrows represent signalling targets and inner bars represent signalling sources in which the colours indicate signalling targets. Irf7 = Interferon regulatory factor 7. Other abbreviations as in Figs. Figs. 1 and 2. *P < 0.05; ***P ≤ 0.001

To investigate impact of experimental DbCM on cardiac cell abundance in context of associated cardiac dysfunction and remodelling, focused analysis of macrophage and monocyte populations was performed across sample groups (Fig. 7A, B). The proportion of MHC-II antigen-presenting macrophages was reduced (*P* = 0.0003), whilst relative abundance of Ccr2^+^ Ly6c^hi^ pro-inflammatory monocytes was increased (*P* = 0.0148) in hearts from HFD/STZ mice compared to control mice. Interestingly, HFD/STZ mice treated with Sacubitril/Valsartan showed suppressed cardiac expansion of Ccr2^+^ Ly6c^hi^ monocytes (*P* = 0.0207) and normalised abundance of MHC-II macrophages (*P* = 0.0113; Fig. 7A, B), although proportion of Ccr2^-^ Ly6c^lo^ monocytes was not different between the HFD/STZ and Sacubitril/Valsartan group (*P* > 0.05; Fig. 7B). These results indicate that Sacubitril/Valsartan prevents infiltration of inflammatory monocytes whilst preserving antigen-presenting macrophages in cardiac tissue in the context of progressive DbCM.

### IRF7 is a central cardiac stress-inducible factor in DbCM

As Sacubitril/Valsartan treatment conferred specific benefits on cardiac inflammation, diastolic function and remodelling in experimental DbCM, subsequent mechanistic analyses focussed on TLF^+^ macrophages, which significantly contribute to ECM deposition and wound healing processes. Differential gene expression analysis revealed Interferon regulatory factor 7 (*Irf7*) amongst the top up-regulated genes in TLF^+^ macrophages in diabetic heart (Fig. 7C). *IRF7* is a master regulator of type I inflammation, which is known to be involved in the pathologically stressed heart, including in DM [27, 28]. To gain broader understanding of transcriptional programmes related to *Irf7*, cell-type-specific gene regulatory networks were constructed in macrophage/monocyte subpopulations using a modified SCENIC pipeline [29, 30]. This analysis identified *Irf7* amongst the highest regulon specificity scores (RSS) in Ccr2^+^ Ly6c^hi^ monocytes and TLF^+^ macrophages in DbCM hearts (Fig. 7D and Supplementary Figure S8). Furthermore, cell–cell communication analysis revealed increased signalling from immune cells to other cardiac cell populations in DbCM mice compared to control, which was suppressed by Sacubitril/Valsartan treatment (Fig. 7E–G). Complementary comparison of information flow indicated that the collagen signalling pathway was prominently activated in HFD/STZ mice and decreased by Sacubitril/Valsartan treatment, particularly signalling from immune cell populations (Fig. 7H–J). Of note, collagen signalling from TLF^+^ macrophages (M2-like phenotype) to fibroblasts was particularly evident in HFD/STZ mice but not in controls or with Sacubitril/Valsartan treatment (Fig. 7H–J). These data indicate that Sacubitril/Valsartan exhibits significant immunomodulatory properties in DbCM hearts, which regulate inflammatory responses through mitigating pathological cellular crosstalk between TLF^+^ macrophages and fibroblasts-related collagen synthesis.

### High glucose condition can induce IRF7 expression in M2-like macrophages and activate collagen synthesis in fibroblasts

**Fig. 8:**
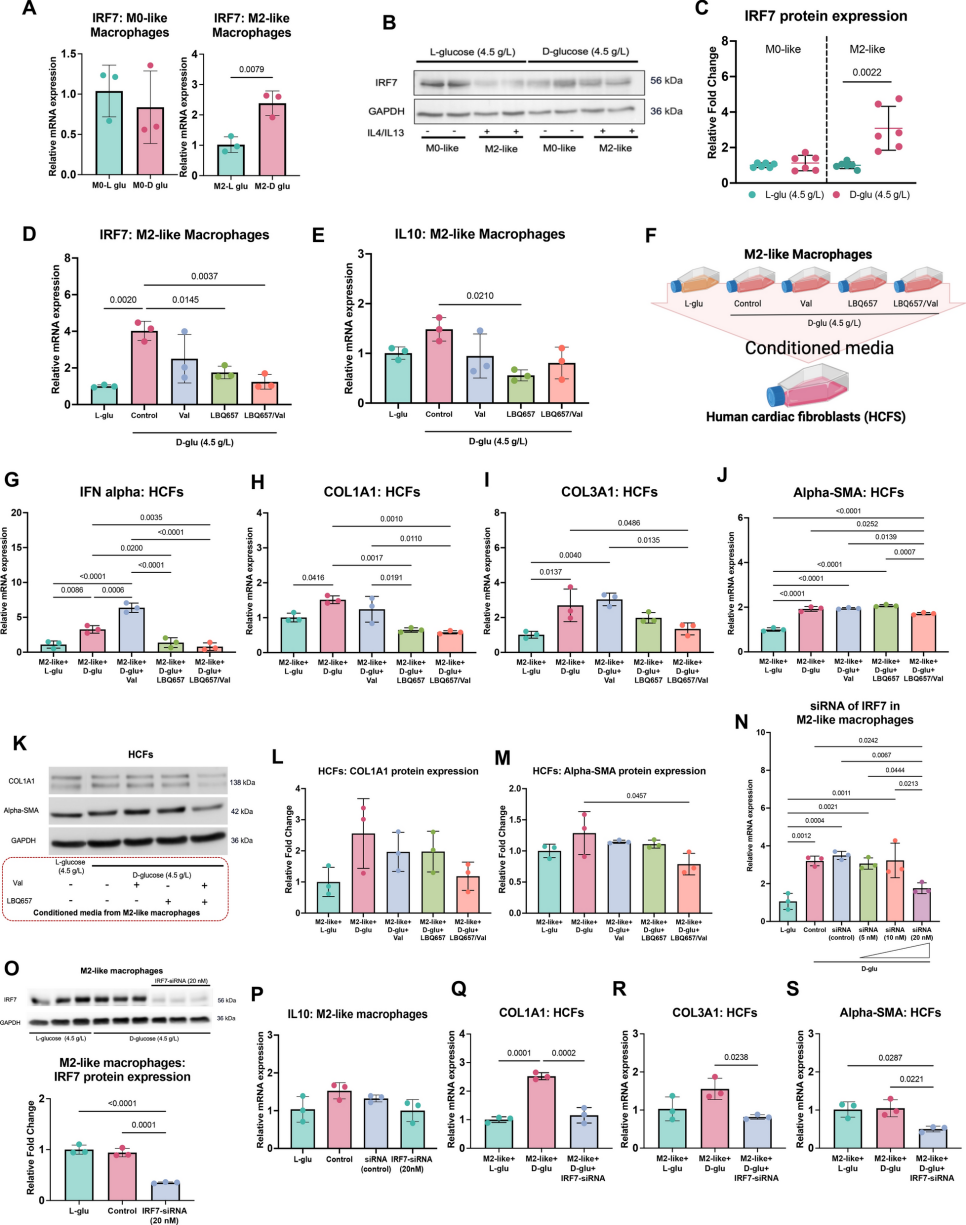
Studies of M2-like macrophages (TLF^+^ macrophages) and fibroblasts. **A**–**C***IRF7* is induced in M2-like macrophages with high D-glucose condition (n = 3 each for RT-qPCR; n = 6 each for Western blot; biological replicates). **D**–**E** Effects of treatments for *IRF7* and *IL10* on M2-like macrophages in glucose conditions (n = 3 each; biological replicates). **F** Experimental design for paracrine interaction between M2-like macrophages and HCFs. **G**–**J** HCFs are treated with conditioned media from M2-like macrophages experiments with or without treatments (n = 3 each; biological replicates). **K**–**M** Western blotting analysis of HCFs treated with conditioned media from M2-like macrophages experiments (n = 3 each; biological replicates). **N** The siRNA of *IRF7* in M2-like macrophages with different concentrations (n = 3 each; biological replicates). **O** Western blotting analysis for IRF7 of siRNA-IRF7 in M2-like macrophages (n = 3 each; biological replicates). **P** Expression of *IL10* of siRNA-IRF7 in M2-like macrophages (n = 3 each; biological replicates). **Q**–**S** HCFs are treated with conditioned media from siRNA of *IRF7* in M2-like macrophages experiments (n = 3 each; biological replicates). Comparison using Unpaired *t* test (**A**, **C**). Comparison using either one-way ANOVA followed by Tukey's multiple comparisons test (**D**–**E**, **G**–**S**). *P* values for one-way ANOVA test < 0.05 for (**D**–**E**, **G**–**J**, **L**–**O**, and **Q**–**S**). If *P* values > 0.05 are not shown in graphs. IRF7 = Interferon regulatory factor 7; IL10 = Interleukin 10 HCFs = Human cardiac fibroblasts; siRNA = Small interfering RNA; LBQ657 = Sacubitrilat (active metabolite of Sacubitril)

Based on the findings of our detailed cell-specific informatic analysis, further in vitro mechanistic investigation focussed on defining the specific role of *IRF7* in M2-like macrophages (TLF^+^ macrophages) and influence on crosstalk with cardiac fibroblasts and collagen production. THP-1 cells were polarised to M2-like pro-repair phenotype, which was confirmed by RT-qPCR based on expression of *MRC1* or *CD206* (cell surface marker), *IL10* and *TGF-β* (secreted factor), and *PPARG* (intracellular marker; Supplementary Figure S9A-E). M2-like macrophages showed induced *IRF7* expression after exposure to high D-glucose compared to L-glucose control (Fig. 8A–C), which was suppressed by treatment with LBQ657 (active metabolite of Sacubitril) either alone or in combination with Valsartan (Fig. 8D) but unaltered in L-glucose controls (Supplementary Figure S9F). As *IRF7* regulates *IL10* linking to anti-inflammatory effects [31], we found that *IL10* was significantly suppressed in high D-glucose with LBQ657, compared to high D-glucose alone (Fig. 8E). HCFs were treated with the conditioned media collected from M2-like macrophages (Fig. 8F). Further analysis revealed that *IFN-alpha* expression was increased in HCFs incubated with conditioned media from high D-glucose and Valsartan treated M2-like macrophages but suppressed in HCFs incubated with conditioned media from both LBQ657 and LBQ657/Val-treated M2-like macrophages in high D-glucose (Fig. 8G). Moreover, *COL1A1* expression was reduced in HCFs treated with conditioned media from high D-glucose M2-like macrophages treated with LBQ657 and LBQ657/Val, compared to high D-glucose alone (Fig. 8H). Similarly, HCFs treated with conditioned media from high D-glucose M2-like macrophages with LBQ657 and LBQ657/Val showed lower expression of *COL3A1*, although only the latter group reached statistical significance when compared to HCFs treated with conditioned media from high D-glucose and Valsartan treated M2-like macrophages (Fig. 8I). HCFs treated with conditioned media from high D-glucose M2-like macrophages with LBQ657/Val showed suppression of *Alpha-SMA* (smooth muscle actin) (Fig. 8J). Western blotting analysis also revealed decreased COL1A1 and Alpha-SMA protein levels in HCFs treated with conditioned media from high D-glucose M2-like macrophages treated with LBQ657/Val (Fig. 8K–M). Collectively, these results indicate that Sacubitril/Valsartan-mediated modulation of *IRF7* expression in pro-repair macrophages alleviates collagen production by mitigating pathological crosstalk with cardiac fibroblasts and fibrogenesis, highlighting a novel mode of action for NEP inhibition in experimental DbCM. The direct role of *IRF7* in this context was verified through gene silencing of *IRF7* in M2-like macrophages using a concentration of 20 nM *IRF7*-siRNA, which markedly reduced *IRF7* expression (Fig. 8N–O), conditioned media from which was used to treat HCFs exposed to high D-glucose and L-glucose control, and the expression of collagen 1 and 3 levels were measured by RT-qPCR. Importantly, these studies showed that expression of *COL1A1, COL3A1, and Alpha-SMA* was normalised in HCFs exposed to high D-glucose and *IRF7*-siRNA conditioned media (Fig. 8Q–S), confirming a likely significant role for *IRF7* signalling in pro-repair macrophages in regulating fibrogenesis, collagen production, and deposition in DbCM.

## Discussion

DM with cardiomyopathy is an important pre-clinical HF stage, which over time can progress into overt HF. It is necessary to shift treatment strategies earlier in the natural history of disease, providing interventions in at-risk populations to prevent or delay HF development [7, 32]. One potential therapeutic target is NEP enzyme, as highlighted in the PARABLE trial of pre-HFpEF patients with a range of CVD risk-factors. NEP enzyme is inhibited by Sacubitril (NEP inhibitor) and elevates several beneficial NEP substrates (such as ANP, BNP, GLP-1) in cardiovascular diseases, suggesting pleiotropic effects of treatment [13, 33–35].

While soluble NEP (sNEP) and NEP activity have been examined in HF patients before [36, 37], we reveal for the first time NEP activity profiling after Sacubitril/Valsartan treatment in a pre-HFpEF population. Our analysis focused on the subset of participants from the PARABLE trial who had a diagnosis of DM. Sacubitril/Valsartan rapidly decreased plasma NEP activity and this early suppression of NEP activity may lead to increased levels of various NEP substrates, providing cardioprotective effects. Elevated plasma NEP activity in diabetic patients was associated with increased LA stiffness index, a prognostic index for progression of HFpEF [38], and diabetic patients treated with Sacubitril/Valsartan suppressed an increase of LA stiffness index at 18 months, suggesting beneficial NEP inhibition. Our DbCM mouse model showed that increasing plasma NEP activity was associated with worsening diastolic function, LV hypertrophy/fibrosis and increasing LV filling pressure, which are hallmarks of DbCM. Sacubitril/Valsartan prevented elevation of plasma NEP activity, ameliorated diastolic dysfunction and cardiac remodelling and deaccelerated DbCM progression. Supportive of these findings is a recent study that demonstrated impaired cardiac function and oxidative stress markers are improved following NEP inhibition in mice with obesity and metabolic heart disease [39]. As expected, our study demonstrated that Sacubitril/Valsartan inhibited NEP activity and mediated GLP-1 increase, improving glycaemic control. Likewise, previous clinical studies showed increased GLP-1 levels in a time-dependent manner and improvement of glycaemic parameters with lower new use of insulin were observed in patients receiving Sacubitril/Valsartan [35, 40]. Our study also demonstrated the benefits of Sacubitril/Valsartan in preventing disease progression in DbCM mice, even in the absence of weight loss, implying that the benefits are independent.

The snRNA-seq was used to investigate DbCM progression and gained insight into the mode of action of Sacubitril/Valsartan in the context of diabetes-induced cardiac impairments. A growing body of evidence supports the role of a systemic pro-inflammatory state, mostly caused by obesity and metabolic stress, as a primary driver of HFpEF pathogenesis [41]. Myocardial inflammation is implicated in developing DbCM and has lately emerged as a pathophysiological factor in cardiac impairments, contributing to hypertrophy, fibrosis, and dysfunction [10]. We studied inflammatory responses in DbCM mice and found that interferon alpha response pathway was a perturbated pathway. These results suggest that DbCM is cardiac inflammation that manifests in diabetic hearts. *IRF7* is a master regulator of type 1 interferon (IFN-alpha/beta)-dependent immune responses, and *IRF7* has been shown previously to be induced in DM [28]. The snRNA-seq data indicated that pro-inflammatory monocytes infiltrated into diabetic hearts, and Sacubitril/Valsartan prevented the influx of these pro-inflammatory cells. In this line, BNP treatment of monocytes in vitro can mitigate the stimulatory effect of monocyte chemoattractant protein-1 (MCP-1) and inhibit monocyte chemotaxis, therefore highlighting a potential mechanism of suppressing monocyte migration from the circulation into injured tissue [42]. During cardiac stress, the current study showed decreases in MHC-II macrophages, which exhibit antigen-processing and -presenting functions, and Sacubitril/Valsartan treatment maintained this MHC-II macrophage population within the diabetic heart, promoting immune surveillance in chronic low-grade inflammation.

M2-like macrophages play significant roles in ECM formation, as well as tissue repair and restoration, and maladaptive immune responses are recognised in the diabetic heart [43, 44]. Based on our cell–cell interaction analysis, TLF^+^ macrophages showed properties of pro-repairing (M2-like phenotype) and linked to fibroblasts, contributing to collagen production and deposition in DbCM mice. These adverse sequelae were alleviated by Sacubitril/Valsartan treatment. Interestingly, in response to chronic inflammation caused by high glucose levels, NEP inhibitor with or without Valsartan modulated *IRF7* expression in M2-like macrophages. These suggest benefits of NEP inhibition, complementary to Valsartan in the context of the diabetic condition. To confirm these beneficial effects, knocked down of *IRF7* in M2-like macrophages and treatment of HCFs with macrophage conditioned media, suppressed collagen 1 and 3 gene expression. Our findings highlight immunomodulatory properties of NEP inhibition added to valsartan in DbCM via modulating cardiac macrophages. Other research in the field supports the concept of targeting *IRF7* in DbCM. In an observational study, *Irf7* was also shown to be increased in cardiac tissue from a model of T1DM [28]. In a mechanistic study, *Irf7* was shown to be involved in the aetiology of metabolic abnormalities in mice after 6 months of HFD, with *Irf7* gene knock out (KO) mice having improved glucose and lipid homeostasis and insulin sensitivity. Although the authors did not examine the heart specifically, they reported that *Irf7 KO* mice on HFD had less macrophage infiltration into multiple organs, preventing local and systemic inflammation [45]. Furthermore, *IRF7* is linked with inflammation and fibrosis in a murine model of experimental sclerosis, and *IRF7* KO reduced profibrotic factors in fibroblasts [46]. However, *IRF7* might have multifunctional roles in other forms of HF. Unlike in DM models, *Irf7* gene expression showed no significant alterations after angiotensin II (AngII)-induced HF, and heart-specific overexpression of *Irf7* significantly attenuated pressure overload–induced cardiac hypertrophy, fibrosis, and dysfunction [47]. As pre-HF patients exhibit great heterogeneity and have various comorbidities, such as hypertension, obesity, and DM, their therapeutic response to Sacubitril/Valsartan may differ, and that the Sacubitril/Valsartan mediated reduction of *Irf7* and improvements in cardiac structure and function reported in this study may be specific to diabetes-induced cardiac dysfunction in pre-HF populations.

Our study has some limitations that need to be acknowledged. First, we did not assess plasma concentrations of other NEP substrates besides GLP-1. These might need to be studied further. Only male mice were used in this study, and any potential differences among sexes have not been reported on. Furthermore, we did not isolate resident cardiac macrophages from experimental DbCM mice to investigate their specific roles related to cardiac remodelling, highlighting an area for future research. Finally, we acknowledge that the small sample size of pre-HF patients with DM in the PARABLE trial limits statistical power, restricting definitive conclusions, particularly for non-significant findings. Larger studies focusing on patients with DbCM with rigorous criteria such as stratified randomisation are needed to minimise conditions associated with compromised clinical outcomes and better understand its uniqueness and the long-term effects of Sacubitril/Valsartan.

## Conclusions

Sacubitril/Valsartan considerably suppresses NEP activity in pre-HFpEF patients with diabetes and in a DbCM mouse model with improving cardiac function and preventing cardiac remodelling. These effects may benefit from the immunomodulatory properties of NEP inhibition added to Valsartan in response to chronic low-grade inflammation, triggered by hyperglycaemia. We also reveal important roles of *IRF7* and add a novel mode of action of Sacubitril/Valsartan via modulating cardiac immune cells in DbCM progression. However, further work is required to study direct effect of other NEP substrates in DbCM.

## Supplementary Information

Additional file1 (DOCX 69293 KB)

## Data Availability

The materials and data that support the findings of this study are available from the corresponding author upon reasonable request.
