# Supplementary material for: Sacubitril/Valsartan attenuates progression of diabetic cardiomyopathy through immunomodulation properties: an opportunity to prevent progressive disease
Source: Cardiovasc Diabetol. 2025 May 14;24:206. doi: 10.1186/s12933-025-02741-5 (PMC12079907; doi:10.1186/s12933-025-02741-5)
Supplement: Supplementary file 1 — Additional file1 (DOCX 69293 KB) [file 12933_2025_2741_MOESM1_ESM.docx]

**Supplementary Materials for**

**Sacubitril/Valsartan Attenuates Progression of Diabetic Cardiomyopathy Through Immunomodulation Properties:**

**An Opportunity to Prevent Progressive Disease**

Narainrit Karuna^1,2^, Lauren Kerrigan^1^, Kevin Edgar^1^, Mark Ledwidge^3^,
Ken McDonald^3^, David J Grieve^1^, Chris J Watson^1,3^

^1^Wellcome-Wolfson Institute for Experimental Medicine, Queen’s University Belfast, Belfast, United Kingdom

^2^Chiang Mai University, Chiang Mai, Thailand

^3^STOP-HF Unit, St. Vincent's University Healthcare Group, Dublin, Ireland

**Corresponding author:** Chris J Watson, [chris.watson@qub.ac.uk](mailto:chris.watson@qub.ac.uk)

Wellcome-Wolfson Institute for Experimental Medicine, Queen’s University Belfast, Belfast, United Kingdom

**DETAILED METHODS**

Mouse echocardiography

Single-nuclei RNA sequencing (snRNA-seq) of DbCM mouse model and downstream analysis

*In vitro* studies

**OTHER SUPPLEMENTARY MATERIAL FOR THIS MANUSCRIPT INCLUDES THE FOLLOWING**

Table S1 Sequences of primers used in RT-qPCR

Table S2 Antibodies and details used in Western blotting

Table S3: Characteristics of pre-HFpEF with type 2 diabetes mellitus of PARABLE trial

Table S4: Characteristics of diabetic cardiomyopathy mouse model

Supplementary Figure S1 Experimental protocol and actual doses of treatments

Supplementary Figure S2 Diabetic cardiomyopathy phenotype before treatments at 12 weeks of study using echocardiography

Supplementary Figure S3 Longitudinal follow-up of effects of treatments on diastolic function (MV E/A and Isovolumic relaxation time)

Supplementary Figure S4 Expression of gene markers for cell population identification

Supplementary Figure S5 Proportions of cells in each sample

Supplementary Figure S6 Subpopulations of macrophages and monocytes

Supplementary Figure S7 Comparison of pathways among subpopulations of monocytes

Supplementary Figure S8 Regulon specificity of Ccr2+ Ly6c(hi) Monocytes

Supplementary Figure S9 In vitro studies of M2-like macrophages and fibroblasts migration assay

The STROBE checklist**Mouse echocardiography**

Mice were anesthetised in a closed chamber with 3% isoflurane in oxygen and imaged using a FUJIFILM Vevo 3100 (VisualSonics, Toronto, Canada) with MX400 probe (20-46 MHz). Mice were in the supine position, and core temperature was maintained at 37 °C with 1.5% isoflurane in oxygen by nose cone during the procedure. Limb electrodes were used to acquire electrocardiogram (ECG). For standard 2D echocardiographic analysis, the parasternal long-axis view was performed to measure LV dimensions and systolic function. Diastolic parameters were assessed using pulsed wave Doppler in the apical 4-chamber view. Left atrial (LA) volume was quantified as previously described [1]. Briefly, the superoinferior (SI) and anteroposterior (AP) dimensions were obtained from the parasternal long-axis view and M-mode, respectively and the mediolateral dimension (ML) was assessed by the parasternal short-axis view. The formula for calculating LA volume was as follows: LA Volume = (4π × SI × AP × ML) / (3 × 2 × 2 × 2). Tracing the border of left atrium was used to quantify left atria area in the apical 4-chamber view, and measurement was performed before opening the mitral valve [2].

**Single-nuclei RNA sequencing (snRNA-seq) of DbCM mouse model and downstream analysis**

**Single nuclei isolation of cardiac samples and processing on the 10X Genomics platform**

Cardiac mouse samples were processed using the previously described method [3]. Library preparation and nuclei sequencing were performed at the Genomics core unit at Queen’s University Belfast. Nuclei suspension was loaded on the 10X Genomics platform with targeted nuclei recovery of 5,000–10,000 per reaction. Libraries for snRNA-seq were generated using the Chromium Single Cell 3′ v2 Reagent Kit (10x Genomics), and sequencing was performed with Illumina Novaseq 6000 sequencer.

**Data pre-processing and Transcriptome mapping**

Sequencing data generated from snRNA-seq libraries were demultiplexed using Cellranger (Version 3.0.2) cellranger mkfastq. Each sample was aligned and mapped to *Mus musculus* reference transcriptome (mm10). Raw gene-barcode matrix outputted by Cellranger was converted into a SingleCellExperiment (SCE) object using the read10xCounts function from the DropletUtils package [4]. Distinguishing droplets containing nuclei from empty droplets was performed by EmptyDrops algorithm with the parameter FDR at 1%, providing filtered gene-cell count matrices for downstream analysis.

**Quality control, batch correction and clustering**

Downstream analysis employed filtered feature-barcode matrices using SingleCellExperiment, scran, and scater packages [5-7]. DecontX package [8] was used to estimate and remove ambient RNA from dataset. Doublets were identified and filtered using scDblFinder package [9]. Performing quality control, snRNA-seq data from individual samples were combined into a single large gene expression matrix and analysed. Single nuclei were filtered out for counts < 100, genes < 1000, and mitochondrial genes > 30%, retaining 62,681 nuclei passing QC. Then, genes were further filtered based on data sparsity. After read count normalization and log transformation, the top 10% of highly variable genes were selected. Principal components were performed to define the appropriate number of principal components for neighbour graph construction. Before manifold construction using t-SNE, batch correction based on matching mutual nearest neighbors technique was performed by Batchelor package [10]. Nuclei were clustered using graph-based clustering, the Louvain algorithm [11]. Differentially expressed genes between each cluster were calculated using the findMarkers function with Welch t-test and considered only genes that were differentially expressed in all pairwise comparisons involving the cluster of interest (Berger and Hsu 1996 (<https://doi.org/10.1214/ss/1032280304)>). Clusters with high similarity in differentially expressed genes were merged. Known cell markers of major cell types were used to distinguish cell clusters, identifying 11 major cell populations. Top 5 differentially expressed genes of each cluster were visualised by dittoSeq package [12].

**Cell type-specific subclustering (subpopulations) analysis**

Macrophages were selected for the second-round of clustering to identify further subpopulations. Next, we re-processed for clustering with the mentioned processes: variance modelling, PCA embedding, graph-based clustering and t-SNE embedding. This subpopulation analysis provided 9 subclustering (k=5), and marker genes for subpopulations were identified using findMarkers function. In macrophages and monocytes, known cell markers were used to identify subpopulations, resulting in 5 different macrophage/monocyte populations. To identify different cluster functions, functional enrichment analysis was performed based on Gene Ontology (GO) databases [13]: biological process (BP), cellular component (CC), and molecular function (MF).

**Differential gene expression analysis**

To examine differentially expressed genes among cell populations, we aggregated cells within a biological replicate (pseudo-bulk) before applying a statistical test. In this scenario, differential expression analysis was performed with edgeR [14] and P-value cut-off of 0.05 was used.

**Pathway enrichment**

Pathway enrichment analysis was performed using SCPA package with default settings [15]. We used a comprehensive gene set list for mouse that included all pathways of the Hallmark and GO databases from Molecular Signatures Database (MSigDB) [16]. Qval translates to larger pathway differences between conditions. A fold change (FC) enrichment score was also calculated when two conditions were provided.

**Single-cell trajectory analysis**

We used Slingshot [17] to investigate lineage reconstruction and pseudotime inference of macrophage and monocyte subsets. This method identifies lineages based on clusters of cells as nodes in a graph and links a minimum spanning tree (MST) between the nodes. The data was previously normalised and clustered; then, the slingshot wrapper function was used to obtain results. Cluster-defining gene expressions were plotted to track changes across different macrophage and monocyte states.

**Gene Regulatory Network inference**

SCENIC (Single-Cell rEgulatory Network Inference and Clustering) package[18] was used to infer transcription factor (TF) networks active in macrophages/monocytes. Analysis was performed using recommended parameters (https://github.com/aertslab/SCENIC) using the mc9nr RcisTarget database. Expression matrix and metadata were extracted from SingleCellExperiment object and transformed into required format for SCENIC pipeline. Then, GENIE3 [19] in the Bioconductor expression matrix was used to identify co-expressed gene modules and infer potential TF targets for each module. Co-expression and DNA motif analyses identified Regulatory modules (regulons), and AUCell package [18] was used to measure their activities. A binary matrix (with 1 for active and 0 for inactive, with threshold determined by the distribution of AUCell scores) was obtained. The top 5 percent of TFs (top5perTarget) were employed to create regulons for each gene.

***In vitro* studies**

Human monocytic THP-1 cells were maintained in Roswell Park Memorial Institute medium (RPMI 1640, Gibco) culture medium containing 10 % of heat-inactivated fetal bovine serum (FBS) (Gibco), 10 mM Hepes (Merck), and 0.05 mM ß-mercaptoethanol (Merck) at a 5% CO2 and 95% air humidified atmosphere. When required, THP-1 monocytes were differentiated into naïve macrophages (M0) by 24 h incubation with 150 nM phorbol 12-myristate 13-acetate (PMA, Sigma, P8139), followed by another 24 h incubation in RPMI medium. M0 macrophages were polarized into M2-like phenotype by incubation with or without IL4 (peproTech) and IL13 (peproTech) at 20 ng/ml for 48 h **(Stage 1 Supplementary Figure S8)**. Then, these cells were treated with or without high glucose conditions (L-glucose and D-glucose at 4.5g/L) in 6-well plates **(Stage 2 Supplementary Figure S8)**. The M2-like phenotypes were confirmed in each stage using RT-qPCR.

M2-like macrophages were cultured in high L-glucose or high D-glucose RPMI and were then treated with valsartan 1μM, LBQ657 1μM, valsartan+LBQ657 1μM, or control for 24 hours. Cell free supernatants were carefully harvested and stored in aliquots at −80°C. Conditioned medium from macrophage culturing was collected and filtered through a 200 nm filter for indirect co-culture with human cardiac fibroblasts (HCFs).

HCF (~0.6 x 10^6) were plated on 6-well plates in low D-glucose Dulbecco’s modified Eagle's minimal essential (DMEM) medium containing 2% FBS, and the conditioned medium containing the cell secretome (CS) of previously treated macrophages were added into each well (50% v/v) and incubated for 24 hours. Cell pellets were collected and stored at −80°C until analysis.

**siRNA of IRF7 in M2-like macrophages**

To study the effects of knockdown of *IRF7* expression in M2-like macrophages, we performed knockdown of *IRF7* in M2-like macrophages using siRNA using ON-TARGETplus Human IRF7 siRNA-smartpool (L-011810-00-0005), obtained from Horizon Discovery, UK. A series of siRNAs (5, 10, and 20 nM) were used in a pilot experiment. 20 nM of *IRF7* siRNA was selected based on significant suppression of *IRF7* at 24 hours*.* M2-like macrophages treated with L-glucose or D-glucose (4.5 g/L) with or without 20 nM *IRF7* siRNA were compared. Cells were collected, and the conditioned media was centrifuged at 1000 RCF for 5 minutes before being filtered with a 200 nm filter and kept at -80°C. This conditioned medium was used to treat HCF. Cell pellets were collected and stored at −80°C until analysis.

**References**

1. Medrano G, Hermosillo-Rodriguez J, Pham T, Granillo A, Hartley CJ, Reddy A, Osuna PM, Entman ML, Taffet GE: **Left Atrial Volume and Pulmonary Artery Diameter Are Noninvasive Measures of Age-Related Diastolic Dysfunction in Mice**. *J Gerontol A Biol Sci Med Sci* 2016, **71**(9):1141-1150.

2. Schnelle M, Catibog N, Zhang M, Nabeebaccus AA, Anderson G, Richards DA, Sawyer G, Zhang X, Toischer K, Hasenfuss G *et al*: **Echocardiographic evaluation of diastolic function in mouse models of heart disease**. *J Mol Cell Cardiol* 2018, **114**:20-28.

3. McLellan MA, Skelly DA, Dona MSI, Squiers GT, Farrugia GE, Gaynor TL, Cohen CD, Pandey R, Diep H, Vinh A *et al*: **High-Resolution Transcriptomic Profiling of the Heart During Chronic Stress Reveals Cellular Drivers of Cardiac Fibrosis and Hypertrophy**. *Circulation* 2020, **142**(15):1448-1463.

4. Lun ATL, Riesenfeld S, Andrews T, Dao TP, Gomes T, participants in the 1st Human Cell Atlas J, Marioni JC: **EmptyDrops: distinguishing cells from empty droplets in droplet-based single-cell RNA sequencing data**. *Genome Biol* 2019, **20**(1):63.

5. Amezquita RA, Lun ATL, Becht E, Carey VJ, Carpp LN, Geistlinger L, Marini F, Rue-Albrecht K, Risso D, Soneson C *et al*: **Orchestrating single-cell analysis with Bioconductor**. *Nat Methods* 2020, **17**(2):137-145.

6. Lun AT, McCarthy DJ, Marioni JC: **A step-by-step workflow for low-level analysis of single-cell RNA-seq data with Bioconductor**. *F1000Res* 2016, **5**:2122.

7. McCarthy DJ, Campbell KR, Lun AT, Wills QF: **Scater: pre-processing, quality control, normalization and visualization of single-cell RNA-seq data in R**. *Bioinformatics* 2017, **33**(8):1179-1186.

8. Yang S, Corbett SE, Koga Y, Wang Z, Johnson WE, Yajima M, Campbell JD: **Decontamination of ambient RNA in single-cell RNA-seq with DecontX**. *Genome Biol* 2020, **21**(1):57.

9. Germain PL, Lun A, Garcia Meixide C, Macnair W, Robinson MD: **Doublet identification in single-cell sequencing data using scDblFinder**. *F1000Res* 2021, **10**:979.

10. Haghverdi L, Lun ATL, Morgan MD, Marioni JC: **Batch effects in single-cell RNA-sequencing data are corrected by matching mutual nearest neighbors**. *Nat Biotechnol* 2018, **36**(5):421-427.

11. Blondel VD, Guillaume J-L, Lambiotte R, Lefebvre E: **Fast unfolding of communities in large networks**. *Journal of Statistical Mechanics: Theory and Experiment* 2008, **2008**(10):P10008.

12. Bunis DG, Andrews J, Fragiadakis GK, Burt TD, Sirota M: **dittoSeq: universal user-friendly single-cell and bulk RNA sequencing visualization toolkit**. *Bioinformatics* 2021, **36**(22-23):5535-5536.

13. Harris MA, Clark J, Ireland A, Lomax J, Ashburner M, Foulger R, Eilbeck K, Lewis S, Marshall B, Mungall C *et al*: **The Gene Ontology (GO) database and informatics resource**. *Nucleic Acids Res* 2004, **32**(Database issue):D258-261.

14. Robinson MD, McCarthy DJ, Smyth GK: **edgeR: a Bioconductor package for differential expression analysis of digital gene expression data**. *Bioinformatics* 2010, **26**(1):139-140.

15. Bibby JA, Agarwal D, Freiwald T, Kunz N, Merle NS, West EE, Singh P, Larochelle A, Chinian F, Mukherjee S *et al*: **Systematic single-cell pathway analysis to characterize early T cell activation**. *Cell Rep* 2022, **41**(8):111697.

16. Liberzon A, Subramanian A, Pinchback R, Thorvaldsdottir H, Tamayo P, Mesirov JP: **Molecular signatures database (MSigDB) 3.0**. *Bioinformatics* 2011, **27**(12):1739-1740.

17. Street K, Risso D, Fletcher RB, Das D, Ngai J, Yosef N, Purdom E, Dudoit S: **Slingshot: cell lineage and pseudotime inference for single-cell transcriptomics**. *BMC Genomics* 2018, **19**(1):477.

18. Aibar S, Gonzalez-Blas CB, Moerman T, Huynh-Thu VA, Imrichova H, Hulselmans G, Rambow F, Marine JC, Geurts P, Aerts J *et al*: **SCENIC: single-cell regulatory network inference and clustering**. *Nat Methods* 2017, **14**(11):1083-1086.

19. Huynh-Thu VA, Irrthum A, Wehenkel L, Geurts P: **Inferring regulatory networks from expression data using tree-based methods**. *PLoS One* 2010, **5**(9).

**Table S1: Sequences of primers used in RT-qPCR**

| **Genes** | **Forward** | **Reverse** | **Species** |
| --- | --- | --- | --- |
| *Irf7* | CTTCAGCACTTTCTTCCGAGA | TGTAGTGTGGTGACCCTTGC | Mouse |
| *Col1a1* | CGATGGATTCCCGTTCGAGT | CGATCTCGTTGGATCCCTGG | Mouse |
| *Col3a1* | CCAAGGGTGCTACTGGACTC | GCTCACCCTTGTTACCGGAT | Mouse |
| *B2m* | TCACACTGAATTCACCCCCA | TCACATGTCTCGATCCCAGT | Mouse |
| *IRF7* | CTTGGCTCCTGAGAGGGCAG | AGCCCAGGCCTTGAAGATG | Human |
| *COL1A1* | CCCCGAGGCTCTGAAGGT | GCAATACCAGGAGCACCATTG | Human |
| *COL3A1* | AGGATGGTTGCACGAAACAC | ACAGCCTTGCGTGTTCGATA | Human |
| *IFN-alpha* | ACTCATACACCAGGTCACGC | CAGTGTAAAGGTGCACATGACG | Human |
| *MRC1* | TGACGAATTGTGGATCGGCT | CCAGTACCCATCCTTGCCTT | Human |
| *IL10* | CAGGGCACCCAGTCTGAGAAC | TGGCAACCCAGGTAACCCTTAAA | Human |
| *TGF-β* | GGAAATTGAGGGCTTTCGCC | CCGGTAGTGAACCCGTTGAT | Human |
| *Alpha-SMA* | GGCTATTCCTTCGTTACTACTGCT | CATCAGGCAACTCGTAACTCTTCTC | Human |
| *PPARG* | GCCTTAACCTCTGCTGGTGA | GTGTCAACCATGGTCATTTCTAAGG | Human |
| *IL1B* | TTCGAGGCACAAGGCACAA | TGGCTGCTTCAGACACTTGAG | Human |
| *IL6* | CCTTCTCCACAAGCGCCTTC | GGAAGGCAGCAGGCAACA | Human |
| *TNF-alpha* | CCCCAGGGACCTCTCTCTAA | GCTTGAGGGTTTGCTACAACA | Human |
| *B2M* | GATGAGTATGCCTGCCGTGT | TGCGGCATCTTCAAACCTCC | Human |

IRF7=Interferon regulatory factor 7; COL1A1= Collagen type I alpha 1 chain; COL3A1= Collagen type III alpha 1 chain; B2M= Beta-2-microglobulin; IFN-alpha= Interferon alpha; MRC1=Mannose receptor C-type 1; IL10=Interleukin 10; TGF-β=Transforming growth factor beta; Alpha-SMA=Alpha smooth muscle actin; PPARG= Peroxisome proliferator activated receptor gamma; IL1B= Interleukin 1 beta; IL6= Interleukin 6; TNF-alpha= Tumor necrosis factor alpha.

**Table S2: Antibodies and details used in Western blotting**

| **Protein** | **Source** | **Cat no** | **Dilution** |
| --- | --- | --- | --- |
| IRF7 | Proteintech | 22392-1-AP | 1:2000 |
| COL1A1 | Cell Signalling Technology | 72026 | 1:1000 |
| Alpha-SMA | Cell Signalling Technology | 19245 | 1:1000 |
| GAPDH | Cell Signalling Technology | 14C10 | 1:5000 |
| Anti-rabbit IgG, HRP-linked | Cell Signalling Technology | 7074 | 1:5000 |

IRF7=Interferon regulatory factor 7; GAPDH= Glyceraldehyde-3-phosphate dehydrogenase

**Table S3: Characteristics of pre-HFpEF with type 2 diabetes mellitus of PARABLE trial**

|  | **Sacubitril/Valsartan** | **Valsartan** | **P-value** | **Overall** |
| --- | --- | --- | --- | --- |
|  | **(N=35)** | **(N=25)** |  | **(N=60)** |
| Gender |  |  |  |  |
| Female | 6 (17.1%) | 8 (32.0%) | 0.3021 | 14 (23.3%) |
| Male | 29 (82.9%) | 17 (68.0%) |  | 46 (76.7%) |
| Age, years |  |  |  |  |
| Median (IQR) | 71.0 (67.5,76.0) | 69.0 (64.0,76.0) | 0.2302 | 71.0 (10) |
| BNP, pg/mL |  |  |  |  |
| Median (IQR) | 63.5 (33.2,103) | 40.8 (28.6,74.0) | 0.0688 | 52.8 (30.1,81.1) |
| NT-proBNP, pg/mL^a^ |  |  |  |  |
| Median (IQR) | 122 (88.5,235) | 111 (61.3,181) | 0.2033 | 119 (84.0,226) |
| Coronary artery disease | 17 (48.6%) | 10 (40.0%) | 0.6930 | 27 (45.0%) |
| Angina | 6 (17.1%) | 1 (4.0%) | 0.2216 | 7 (11.7%) |
| Myocardial infarction | 5 (14.3%) | 3 (12.0%) | >0.9999 | 8 (13.3%) |
| Ischemic heart disease | 17 (48.6%) | 10 (40.0%) | 0.6930 | 27 (45.0%) |
| Deep vein thrombosis | 1 (2.9%) | 0 (0%) | - | 1 (1.7%) |
| Pulmonary embolism | 0 (0%) | 1 (4.0%) | - | 1 (1.7%) |
| Peripheral vascular disease | 2 (5.7%) | 0 (0%) | - | 2 (3.3%) |
| Atrial fibrillation | 4 (11.4%) | 1 (4.0%) | 0.3899 | 5 (8.3%) |
| Other arrhythmia diseases | 26 (74.3%) | 7 (28.0%) | 0.0009 | 33 (55.0%) |
| Hypertension | 33 (94.3%) | 23 (92.0%) | >0.9999 | 56 (93.3%) |
| Dyslipidaemia | 31 (88.6%) | 23 (92.0%) | >0.9999 | 54 (90.0%) |
| Stroke | 2 (5.7%) | 0 (0%) | - | 2 (3.3%) |
| Transient ischaemic attack | 1 (2.9%) | 0 (0%) | - | 1 (1.7%) |
| Aortic aneurysm | 1 (2.9%) | 0 (0%) | - | 1 (1.7%) |
| Chronic obstructive pulmonary disease | 2 (5.7%) | 2 (8.0%) | 0.1590 | 4 (6.7%) |
| Chronic kidney disease | 14 (40.0%) | 5 (20.0%) | 0.1737 | 19 (31.7%) |
| Obesity | 16 (45.7%) | 17 (68.0%) | 0.1478 | 33 (55.0%) |
| Vascular diseases | 20 (57.1%) | 11 (44.0%) | 0.4579 | 31 (51.7%) |
| Benzodiazepines | 2 (5.7%) | 1 (4.0%) | >0.9999 | 3 (5.0%) |
| Antiarrhythmic drugs | 1 (2.9%) | 1 (4.0%) | >0.9999 | 2 (3.3%) |
| Alpha-blockers | 7 (20.0%) | 8 (32.0%) | 0.4497 | 15 (25.0%) |
| Beta-blockers | 20 (57.1%) | 11 (44.0%) | 0.4579 | 31 (51.7%) |
| Calcium-channel blockers | 16 (45.7%) | 8 (32.0%) | 0.4227 | 24 (40.0%) |
| Aldosterone | 3 (8.6%) | 2 (8.0%) | >0.9999 | 5 (8.3%) |
| Statin | 26 (74.3%) | 22 (88.0%) | 0.3261 | 48 (80.0%) |
| Other dyslipidaemia drugs | 1 (2.9%) | 2 (8.0%) | 0.5653 | 3 (5.0%) |
| Thiazide diuretics | 13 (37.1%) | 9 (36.0%) | 0.9278 | 22 (36.7%) |
| Loop diuretics | 3 (8.6%) | 2 (8.0%) | >0.9999 | 5 (8.3%) |
| Antiplatelet drugs (no aspirin) | 4 (11.4%) | 0 (0%) | - | 4 (6.7%) |
| Aspirin | 24 (68.6%) | 17 (68.0%) | 0.8146 | 41 (68.3%) |
| Anticoagulant drugs (no warfarin) | 4 (11.4%) | 1 (4.0%) | 0.3899 | 5 (8.3%) |
| Insulin | 6 (17.1%) | 5 (20.0%) | >0.9999 | 11 (18.3%) |
| Any oral antidiabetic drugs | 28 (80.0%) | 23 (92.0%) | 0.2815 | 51 (85.0%) |
| Metformin | 25 (71.4%) | 20 (80.0%) | 0.6501 | 45 (75.0%) |
| Sulfonylureas | 7 (20.0%) | 7 (28.0%) | 0.6798 | 14 (23.3%) |
| DPP-4 inhibitors | 4 (11.4%) | 5 (20.0%) | 0.4697 | 9 (15.0%) |
| GLP-1 agonists | 0 (0%) | 1 (4.0%) | - | 1 (1.7%) |
| SGLT-2 inhibitors | 2 (5.7%) | 1 (4.0%) | >0.9999 | 3 (5.0%) |
| Thiazolidinediones | 1 (2.9%) | 0 (0%) | - | 1 (1.7%) |

Statistical tests for differences on continuous variables between groups were Student t‐test or Wilcoxon signed–rank test. The Shapiro–Wilk test was used to test whether variables could be considered normally distributed with alpha set at 0.05. Statistical tests for differences on binary variables between groups were the Chi‐squared test or Fisher’s exact test, depending on whether the expected frequency observations in the 2 × 2 summary table were less than 5. BNP=B-type natriuretic peptide; NT-proBNP=N terminal pro B-type natriuretic peptide; DDP-4=Dipeptidyl peptidase-4; GLP-1=Glucagon-like peptide-1; SGLT-2= Sodium-glucose cotransporter-2. ^a^ Missing 1 from the valsartan group.

**Table S4: Characteristics of diabetic cardiomyopathy mouse model at 24 weeks of study**

|  | **P values for ANOVA/ Kruskal-Wallis** | **CD** | **HFD/STZ** | **HFD/STZ +**  **Sac/Val** | **HFD/STZ +**  **Val** |
| --- | --- | --- | --- | --- | --- |
| Weight (g) | 0.0001 | 31.67±2.84 | 39.00±4.38* | 41.90±6.03* | 37.17±4.63* |
| HW (mg) | 0.1467 | 205.0±50.18 | 260.9±84.79 | 194.3±21.49 | 185.0±32.89 |
| HW/TL (mg/mm) | 0.0002 | 9.880±2.33 | 14.55±3.78* | 9.609±1.54^#^ | 10.57±1.65^#^ |
| SBP (mmHg) | 0.6433 | 108.9±5.86 | 109.5±7.34 | 105.9±5.68 | 108.6±6.54 |
| HbA1C (%) | <0.0001 | 4.508±0.28 | 5.964±1.22* | 5.322±0.52* | 5.975±1.24* |
| FBG (mmol/L) | <0.0001 | 7.733±1.16 | 15.78±5.32* | 11.20±2.48 | 15.22±6.13* |
| QUICKI | <0.0001 | 0.2509± 0.01 | 0.2253± 0.01* | 0.2249± 0.01* | 0.2357± 0.01* |
| HOMA‐β | 0.0019 | 401.9± 228.40 | 218.6± 146.10 | 462.3± 274.80^#$^ | 178.8± 104.90* |

CD, n =12; HFD/STZ, n = 11; HFD/STZ + Sac/Val, n = 7-10; HFD/STZ + Sac/Val, n = 12. HW=Heart weight; TL=Tibia length; SBP=Systolic blood pressure; FBG=Fasting blood glucose; QUICKI=Quantitative insulin sensitivity check index; HOMA‐β=Homeostasis model assessment of β-cell function. Values are the mean±SD. If P values > 0.05 are not shown in graphs. n=Number of mice (biological replicates). Comparison using either one-way ANOVA followed by Tukey's multiple comparisons test or Kruskal-Wallis test followed by Dunn's multiple comparisons test. CD=Control diet; HFD/STZ=High fat diet/streptozotocin; Sac/Val=Sacubitril/Valsartan; Val=Valsartan. *P < 0.05 compared to CD; ^#^P < 0.05 compared to HFD/STZ; ^$^P< 0.05 compared to HFD/STZ + Val.

**Supplementary Figure S1 Experimental protocol and actual doses of treatments**


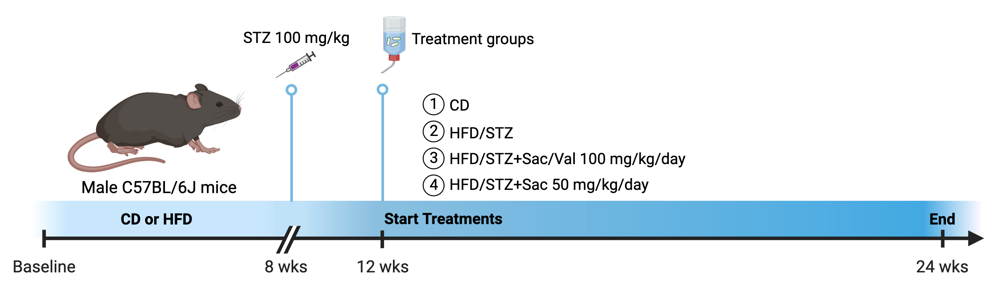


**A**

**B**

(A) Experimental design for DbCM mouse model using HFD/STZ then assigned to treatments. Images generated by Biorender.com. Image usage is covered by BioRender’s Academic License Terms. (B) Actual doses of Sacubitril/Valsartan (n=10) and Valsartan (n=12) over the 12-week course of treatment period. n=Number of mice (biological replicates). DbCM=Diabetic cardiomyopathy; BPM=Beat per minute; CD=Control diet; HFD=High-fat diet; STZ=Streptozotocin; Sac/Val=Sacubitril/Valsartan; Val=Valsartan.

**Figure S2 Diabetic cardiomyopathy phenotype at 12 weeks of study using echocardiography**

**A**

**B**

**C**

**D**

**E**

**F**

**G**

**H**

**I**

**J**

**K**

**L**


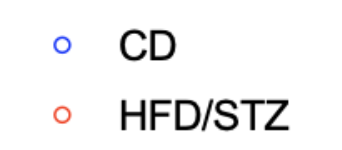


(A) Ejection fraction. (B) Fractional shortening. (C) Left ventricular diameter in systolic phase. (D) Left ventricular diameter in diastolic phase. (E) Left ventricular volume in systolic phase. (F) Left ventricular volume in diastolic phase. (G) Left ventricular anterior wall in systolic phase. (H) Left ventricular anterior wall in diastolic phase. (I) Left ventricular posterior wall in systolic phase. (J) Left ventricular posterior wall in diastolic phase. (K) Ratio of the early (E-wave) to late (A-wave) ventricular filling velocities (L) Isovolumic relaxation time. Comparison using either Unpaired t test or Mann Whitney test. CD n=12; HFD/STZ n=35-36. n=Number of mice (biological replicates). CD=Control diet; HFD/STZ=High fat diet/streptozotocin. *P<0.05 and **P≤0.01.

**Figure S3 Longitudinal follow-up of effects of treatments on
diastolic function (MV E/A and** **Isovolumic relaxation time)**


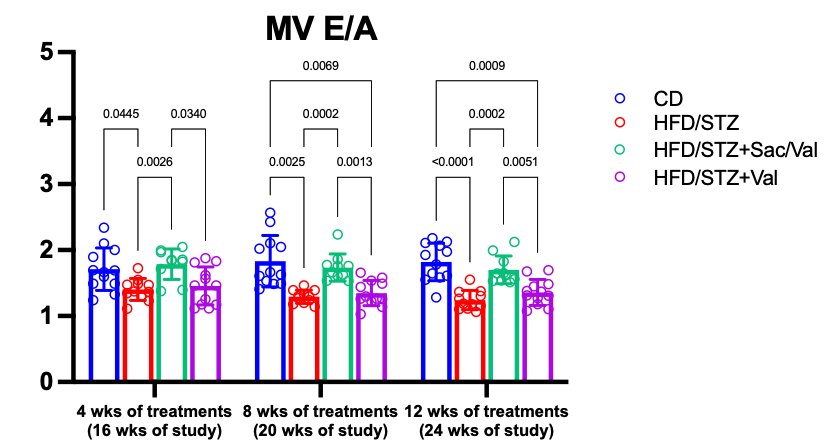

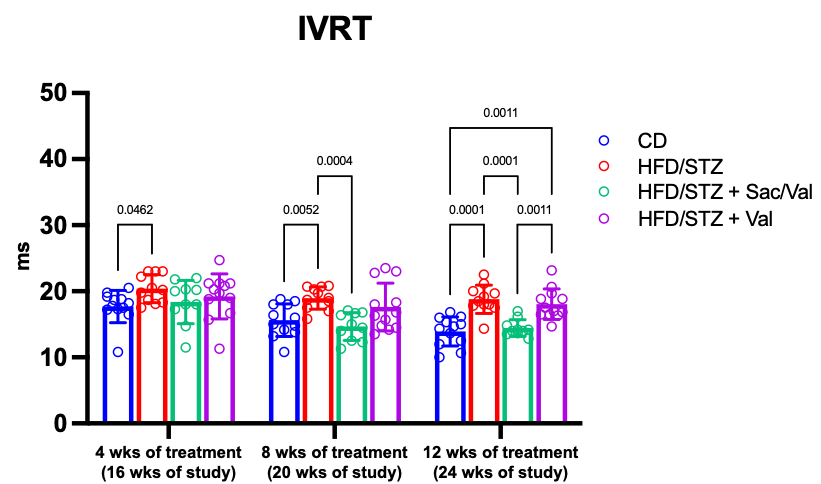


**A**

**B**

(A) Ratio of the early (E-wave) to late (A-wave) ventricular filling velocities after treatments and (B) Isovolumic relaxation time after treatments. Comparison using mixed model ANOVA followed by Tukey's multiple comparisons test. CD n=12; HFD/STZ n=11; HFD/STZ + Sac/Val n=10; HFD/STZ + Val n=12. n=Number of mice (biological replicates). CD=Control diet; HFD/STZ=High fat diet/streptozotocin; Sac/Val=Sacubitril/Valsartan; Val=Valsartan.

**Figure S4 Expression of markers genes for cell population**


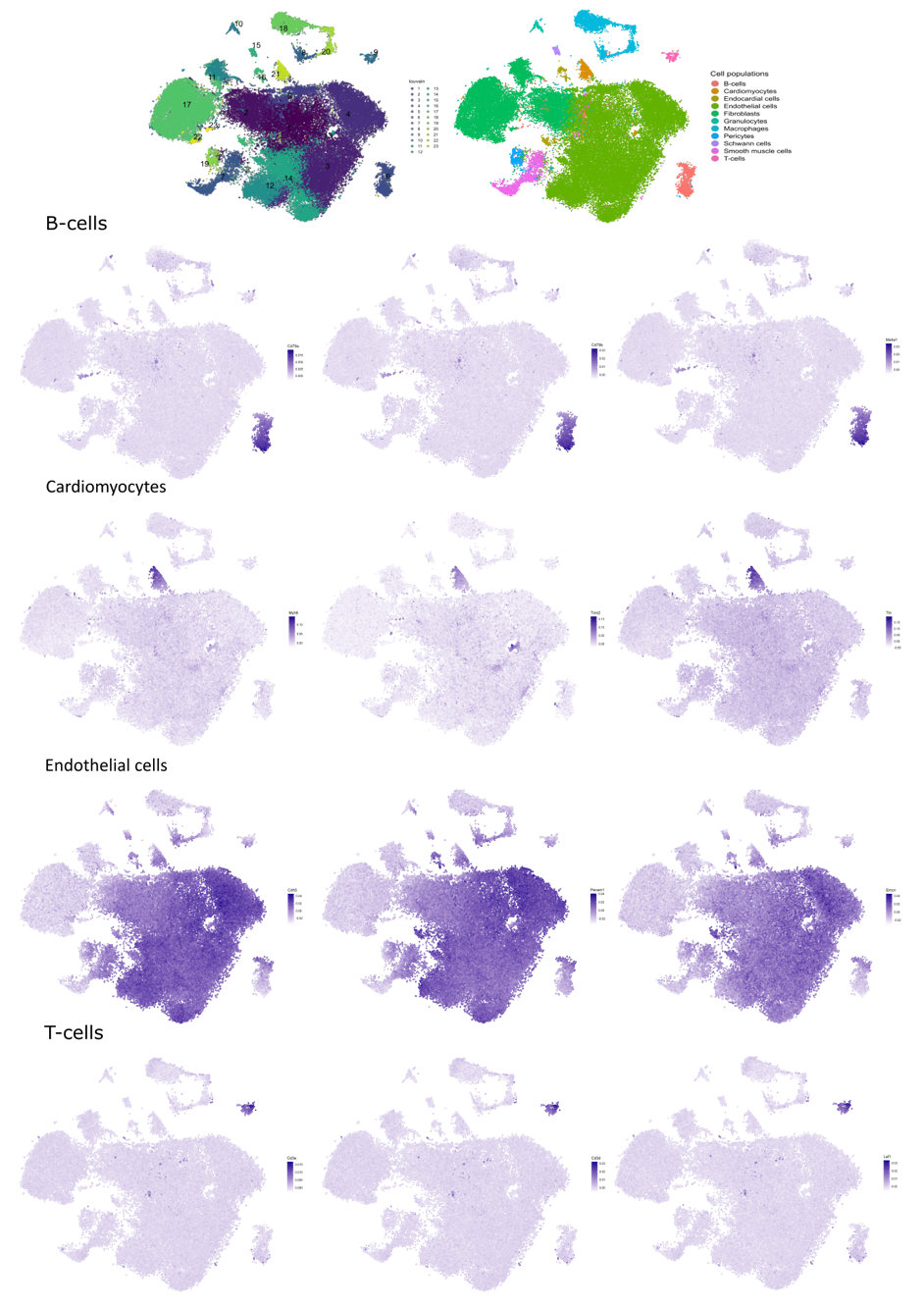


Cd79a

Cd79b

Ms4a1

Myh6

Tnnt2

Ttn

Cdh5

Pecam1

Emcn

Cd3e

Cd3d

Lef1

**A**

**B**

**C**

**D**

**E**

**F**

(A) Louvain clustering. (B) Major cell populations. (C) Marker genes for B-cells (*Cd79a*, *Cd79b*, and *Ms4a1*). (D) Marker genes for cardiomyocytes (*Myh6*, *Tnnt2*, and *Ttn*). (E) Marker genes for endothelial cells (*Cdh5*, *Pecam1*, and *Emcn*). (F) Marker genes for T-cells (*Cd3e*, *Cd3d*, and *Lef1*).

**Figure S4 Expression of markers genes for cell population (cont.)**


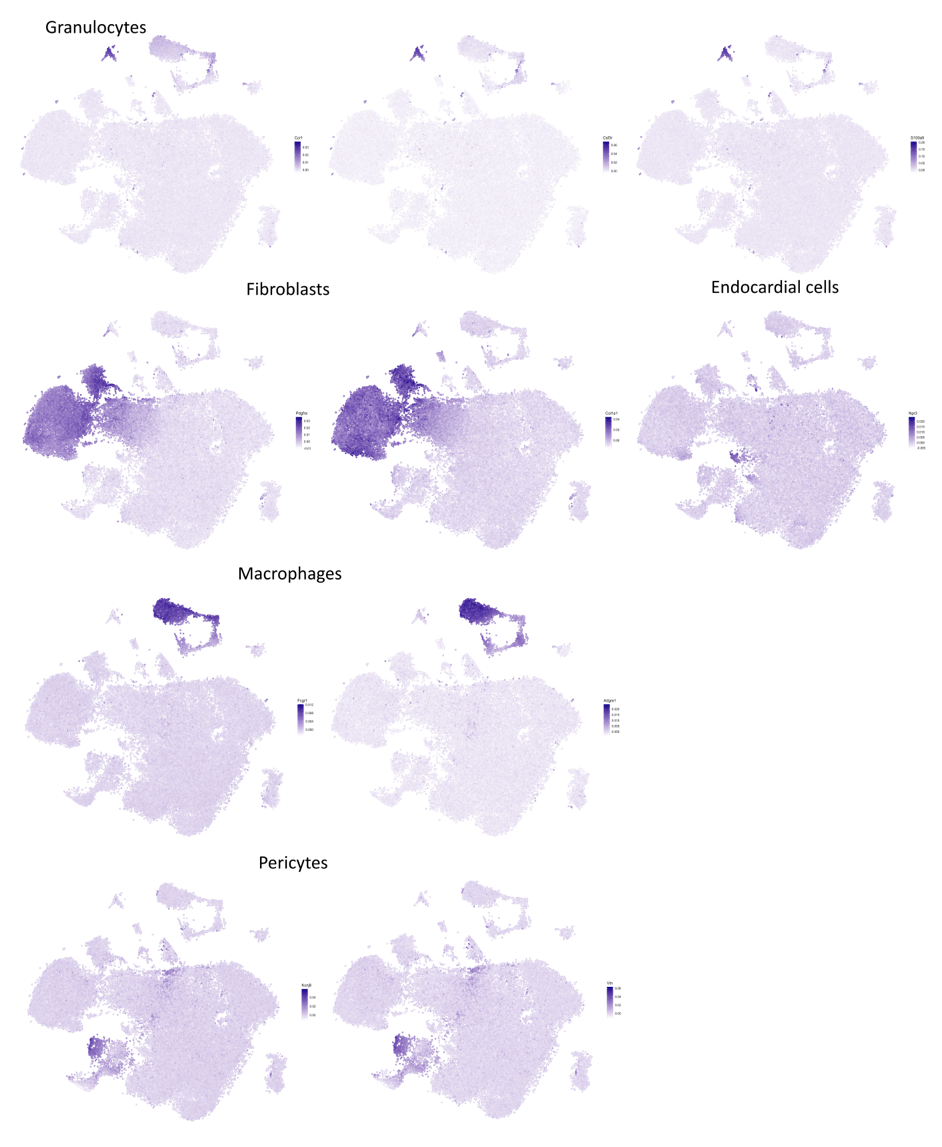


Ccr1

Csf3r

S100a9

Pdgfra

Col1a1

Npr3

Fcgr1

Adgre1

Kcnj8

Vtn

**G**

**H**

**I**

**J**

**K**

(G) Marker genes for granulocytes (*Ccr1*, *Csf3r*, and *S100a9*). (H) Marker genes for fibroblasts (*Pdgfra*, *Col1a1*). (I) Marker gene for endocardial cells (*Npr3*). (J) Marker genes for macrophages (*Fcgr1* and *Adgre1*). (K) Marker genes for pericytes (*Kcnj8* and *Vtn*).

**Figure S4 Expression of markers genes for cell population (cont.)**


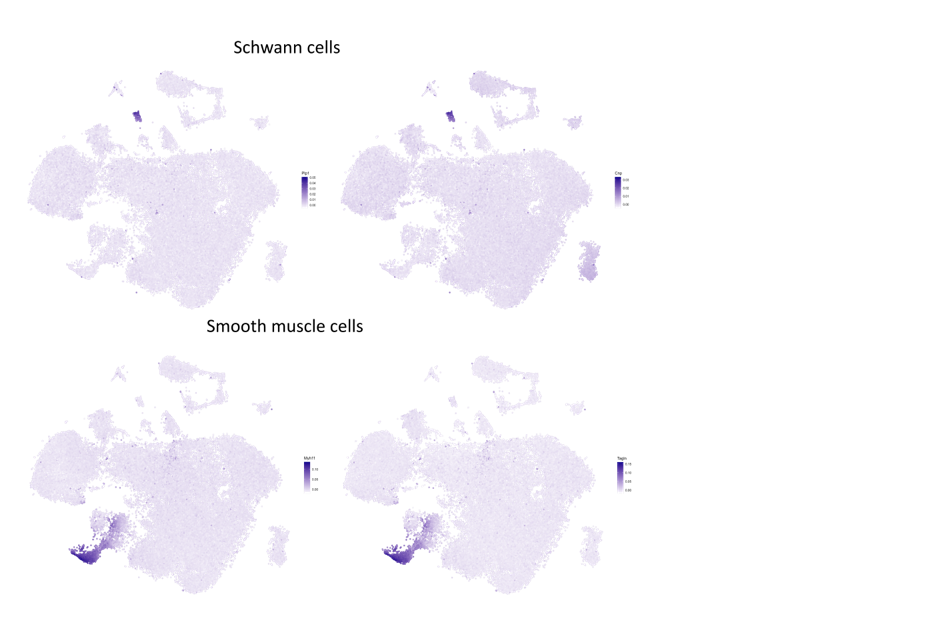


Plp1

Cnp

Tagln

Myh11

**L**

**M**

(L) Marker genes for Schwann cells (*Plp1* and *Cnp*). (M) Marker genes for smooth muscle cells (*Tagln* and *Myh11*).

**Figure S5** **Proportions of cells in each sample**


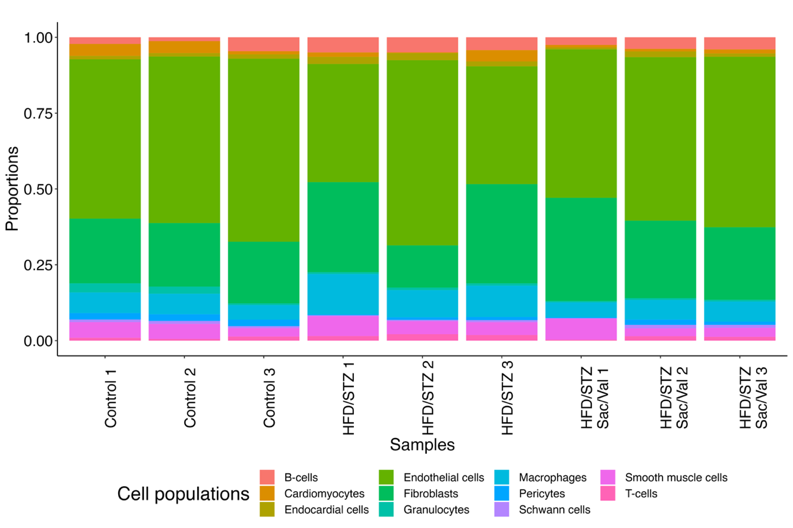


Cell proportions across samples. HFD/STZ=High fat diet/streptozotocin; Sac/Val=Sacubitril/Valsartan.

**Figure S6 Subpopulations of macrophages and monocytes**


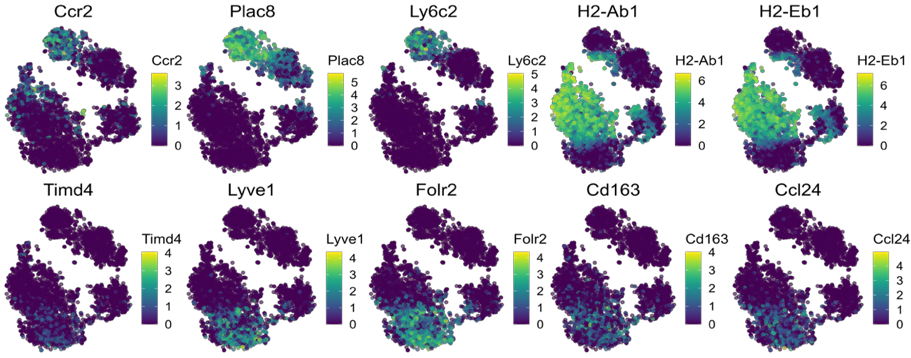

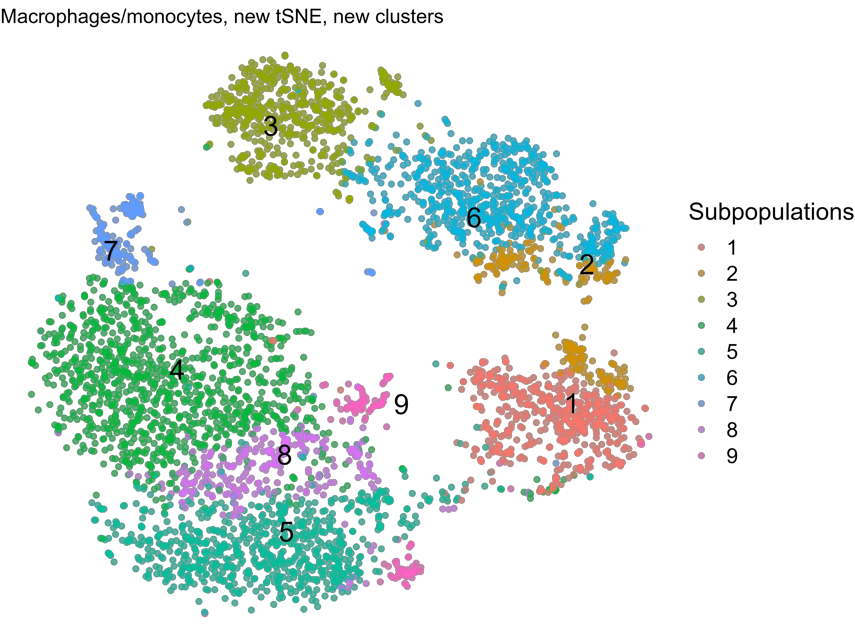


A


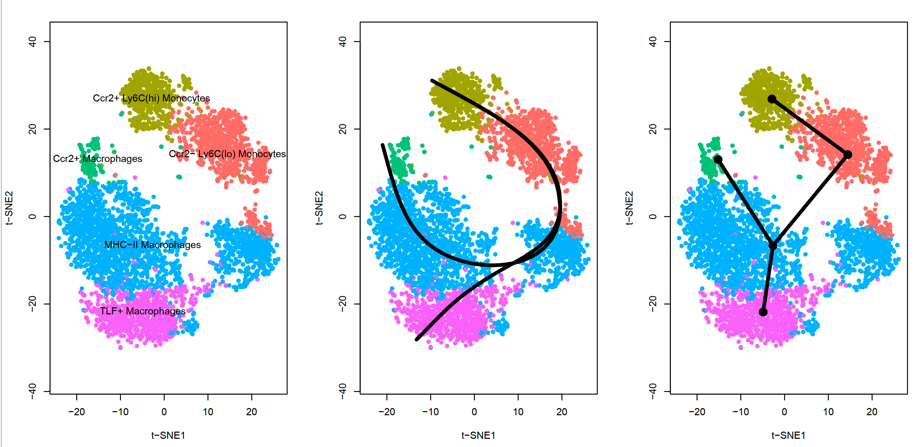


B

C


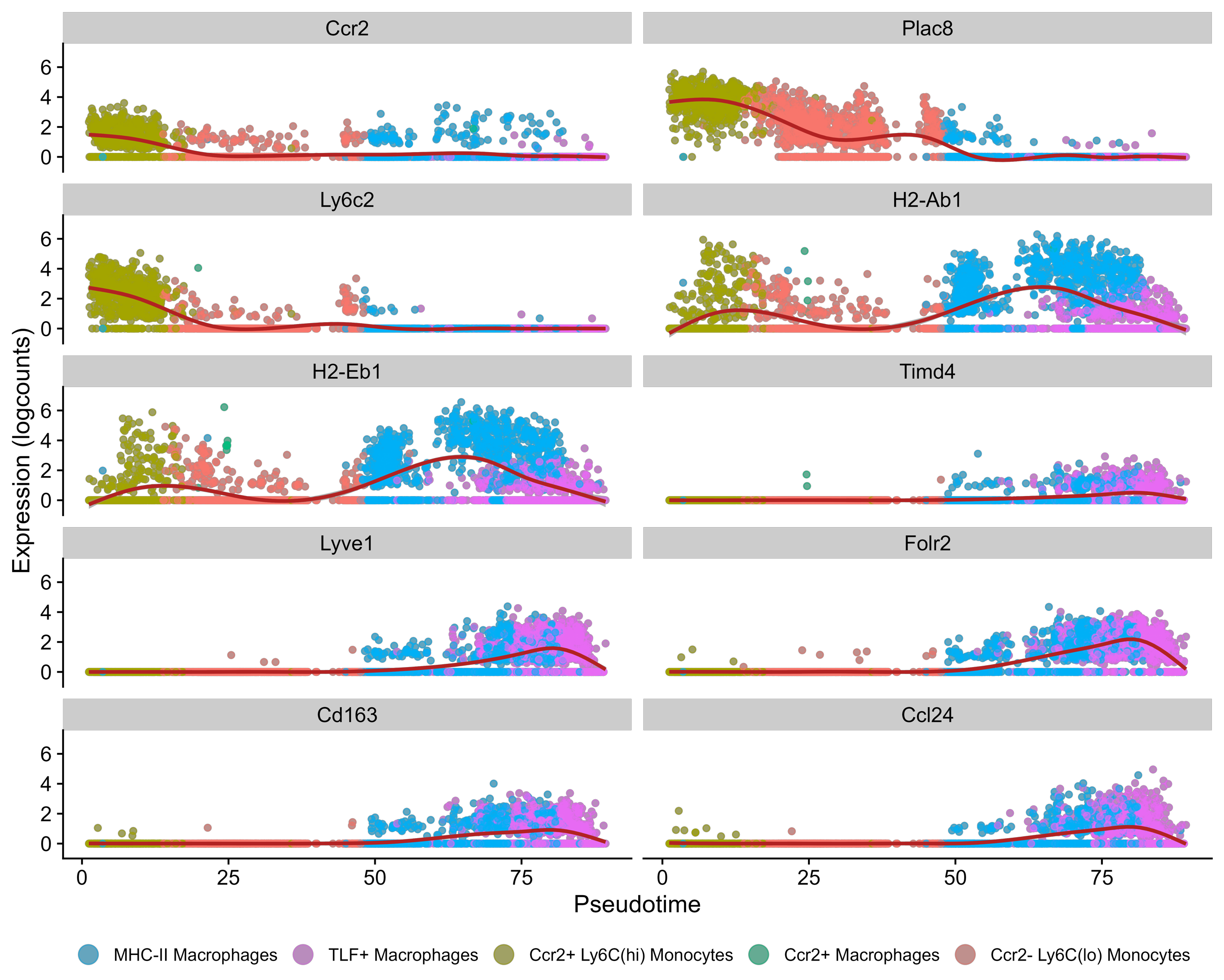


D

(A) Louvain clustering for subpopulation of macrophages and monocytes. (B) Marker genes for macrophages and monocytes (*Ccr2*, *Plac8*, *Ly6c2*, *H2-Ab1*, *H2-Eb1*, *Timd4*, *Lyve1*, *Folr2*, *Cd163*, and *Ccl24*). (C) Pseudotime analysis of subpopulation of macrophages and monocytes. (D) Marker genes for subpopulation of macrophages and monocytes on pseudotime.

**Figure S7 Comparison of pathways among subpopulations of monocytes**
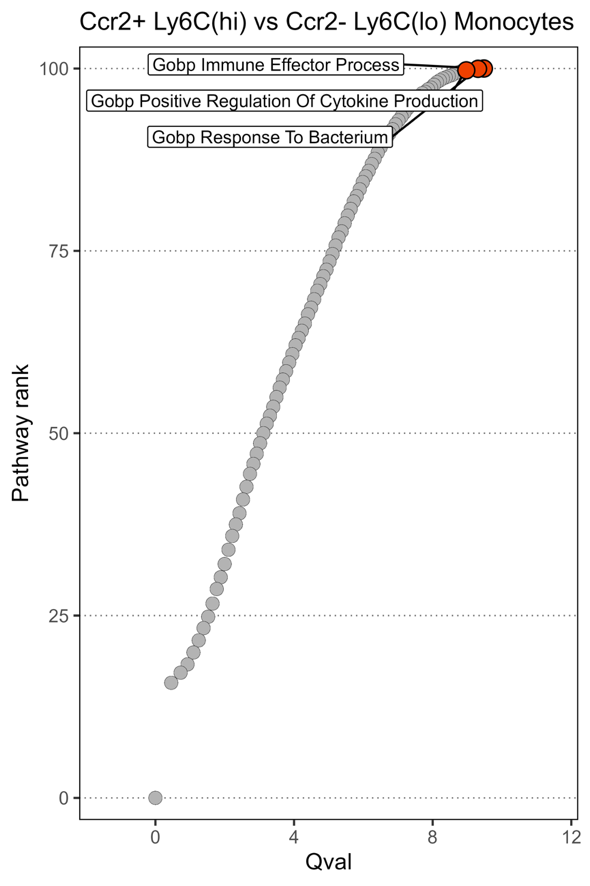


Enriched pathways comparing Ccr^2+^ Ly6C^(hi)^ monocytes to Ccr^2-^ Ly6C^(lo)^ monocytes. Qval indicates the size of distribution change for pathways.

**Figure S8 Regulon specificity of Ccr2+ Ly6c(hi) Monocytes**

**
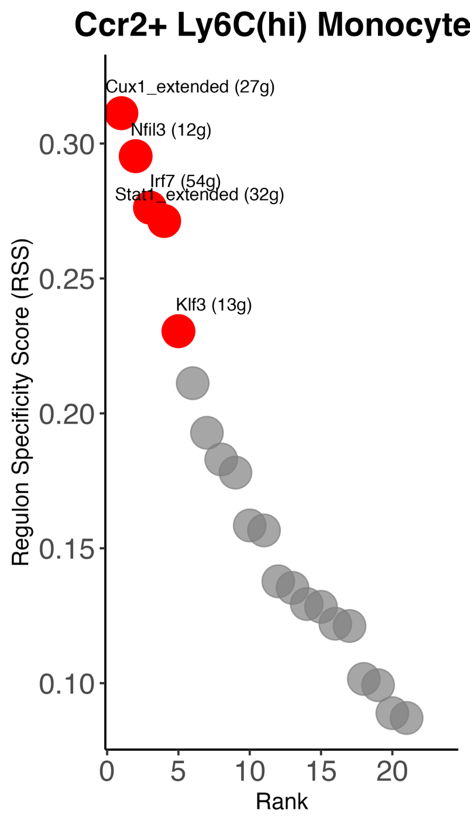
**

Regulon specificity score (RSS) of Ccr^2+^ Ly6c^(hi)^ monocytes by SCENIC pipeline.

**Figure S9 *In vitro* studies of M2-like macrophages and fibroblasts migration assay**


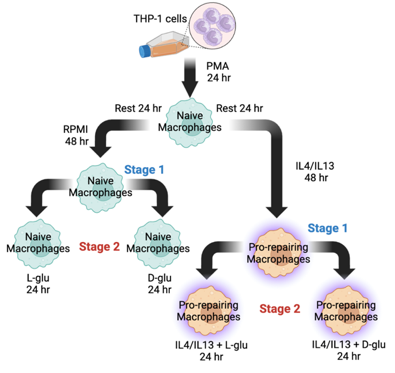


A

B

C

D

E

F


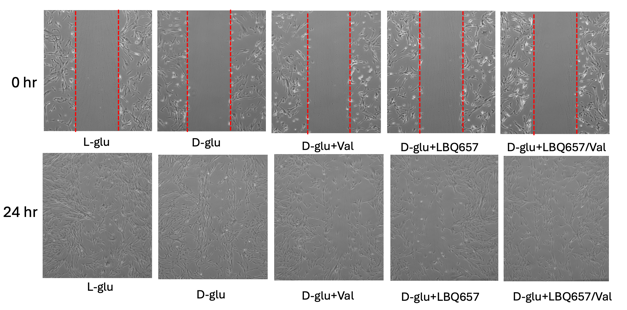


G

H

(A) Design of *in vitro* studies. (B-C) Marker genes for M1-like macrophage (*IL1b*, *IL6*, and *TNF-alpha*) and M2-like macrophage (*MRC1*, *IL10*, *TGF-β* and *PPARG*), n=3 (biological replicates). (D-E) Marker genes treated with high L-glucose or high D-glucose for M1-like macrophage (*IL1b*, *IL6*, and *TNF-alpha*) and M2-like macrophage (*MRC1*, *IL10*, *TGF-β* and *PPARG*), n=3 (biological replicates). (F) Expression of *IRF7* in M2-like macrophages in high D-glucose or high L-glucose with Val, LBQ657, and LBQ657/Val, n=3 (biological replicates). (G-H) Migration assay of fibroblasts treated with conditioned media from M2-like macrophage experiments at 0 hour and 24 hours. Comparison using either Unpaired t test or Mann Whitney test (B-E). Comparison using either one-way ANOVA followed by Tukey's multiple comparisons test (F) or Kruskal-Wallis test followed by Dunn's multiple comparisons test. P values for one-way ANOVA test < 0.05 for (F). If P values >0.05 are not shown in graphs. IRF7=Interferon regulatory factor 7; Val=Valsartan; LBQ657=Sacubitrilat (active metabolite of Sacubitril).

STROBE Statement—Checklist of items that should be included in reports of ***cohort studies***

|  | Item No | Recommendation | Page No |
| --- | --- | --- | --- |
| **Title and abstract** | 1 | (*a*) Indicate the study’s design with a commonly used term in the title or the abstract | 1-2 |
|  |  | (*b*) Provide in the abstract an informative and balanced summary of what was done and what was found | 2 |
| Introduction | | | |
| Background/rationale | 2 | Explain the scientific background and rationale for the investigation being reported | 3 |
| Objectives | 3 | State specific objectives, including any prespecified hypotheses | 3 |
| Methods | | | |
| Study design | 4 | Present key elements of study design early in the paper | 4-7 |
| Setting | 5 | Describe the setting, locations, and relevant dates, including periods of recruitment, exposure, follow-up, and data collection | 4 |
| Participants | 6 | (*a*) Give the eligibility criteria, and the sources and methods of selection of participants. Describe methods of follow-up | 4 |
|  |  | (*b*) For matched studies, give matching criteria and number of exposed and unexposed | N/A |
| Variables | 7 | Clearly define all outcomes, exposures, predictors, potential confounders, and effect modifiers. Give diagnostic criteria, if applicable | 4 |
| Data sources/ measurement | 8* | For each variable of interest, give sources of data and details of methods of assessment (measurement). Describe comparability of assessment methods if there is more than one group | 4-7, Supplementary methods |
| Bias | 9 | Describe any efforts to address potential sources of bias | 4 |
| Study size | 10 | Explain how the study size was arrived at | N/A |
| Quantitative variables | 11 | Explain how quantitative variables were handled in the analyses. If applicable, describe which groupings were chosen and why | 4,7, Supplementary methods |
| Statistical methods | 12 | (*a*) Describe all statistical methods, including those used to control for confounding | 7, Supplementary methods |
|  |  | (*b*) Describe any methods used to examine subgroups and interactions | 7, Supplementary methods |
|  |  | (*c*) Explain how missing data were addressed | Supplementary methods |
|  |  | (*d*) If applicable, explain how loss to follow-up was addressed | N/A |
|  |  | (*e*) Describe any sensitivity analyses | N/A |
| Results | | |  |
| Participants | 13* | (a) Report numbers of individuals at each stage of study—eg numbers potentially eligible, examined for eligibility, confirmed eligible, included in the study, completing follow-up, and analysed | 7-13 |
|  |  | (b) Give reasons for non-participation at each stage | N/A |
|  |  | (c) Consider use of a flow diagram | N/A |
| Descriptive data | 14* | (a) Give characteristics of study participants (eg demographic, clinical, social) and information on exposures and potential confounders | Supplementary Table S3 |
|  |  | (b) Indicate number of participants with missing data for each variable of interest | 7 |
|  |  | (c) Summarise follow-up time (eg, average and total amount) | N/A |
| Outcome data | 15* | Report numbers of outcome events or summary measures over time |  |

| Main results | 16 | (*a*) Give unadjusted estimates and, if applicable, confounder-adjusted estimates and their precision (eg, 95% confidence interval). Make clear which confounders were adjusted for and why they were included | N/A |
| --- | --- | --- | --- |
|  |  | (*b*) Report category boundaries when continuous variables were categorized | N/A |
|  |  | (*c*) If relevant, consider translating estimates of relative risk into absolute risk for a meaningful time period | N/A |
| Other analyses | 17 | Report other analyses done—eg analyses of subgroups and interactions, and sensitivity analyses | N/A |
| Discussion | | | |
| Key results | 18 | Summarise key results with reference to study objectives | 13-15 |
| Limitations | 19 | Discuss limitations of the study, taking into account sources of potential bias or imprecision. Discuss both direction and magnitude of any potential bias | 13-15 |
| Interpretation | 20 | Give a cautious overall interpretation of results considering objectives, limitations, multiplicity of analyses, results from similar studies, and other relevant evidence | 13-15 |
| Generalisability | 21 | Discuss the generalisability (external validity) of the study results | 13-15 |
| Other information | | | |
| Funding | 22 | Give the source of funding and the role of the funders for the present study and, if applicable, for the original study on which the present article is based | 16 |

*Give information separately for exposed and unexposed groups.

**Note:** An Explanation and Elaboration article discusses each checklist item and gives methodological background and published examples of transparent reporting. The STROBE checklist is best used in conjunction with this article (freely available on the Web sites of PLoS Medicine at http://www.plosmedicine.org/, Annals of Internal Medicine at http://www.annals.org/, and Epidemiology at http://www.epidem.com/). Information on the STROBE Initiative is available at http://www.strobe-statement.org.
